# *Limosilactobacillus reuteri* 6475 and Prevention of Early Postmenopausal Bone Loss: A Randomized Clinical Trial

**DOI:** 10.1001/jamanetworkopen.2024.15455

**Published:** 2024-06-12

**Authors:** Giulia Gregori, Aldina Pivodic, Per Magnusson, Lisa Johansson, Ulrika Hjertonsson, Emma Brättemark, Mattias Lorentzon

**Affiliations:** 1 Sahlgrenska Osteoporosis Centre, Department of Internal Medicine and Clinical Nutrition, Institute of Medicine, University of Gothenburg, Gothenburg, Sweden; 2 APNC, Gothenburg, Sweden; 3 Department of Ophthalmology, Sahlgrenska University Hospital, the Västra Götaland Region, Mölndal, Sweden; 4 Department of Clinical Chemistry, and Department of Biomedical and Clinical Sciences, Linköping University, Linköping, Sweden; 5 The Västra Götaland Region, Department of Orthopedics, Sahlgrenska University Hospital, Mölndal, Sweden; 6 Geriatric Medicine, Sahlgrenska University Hospital, the Västra Götaland Region, Mölndal, Sweden; 7 Mary McKillop Institute for Health Research, Australian Catholic University, Melbourne, Australia

## Abstract

**Importance:**

Daily supplementation with the probiotic *Limosilactobacillus reuteri* ATCC PTA 6475 (*L reuteri*) vs placebo has previously been demonstrated to reduce bone loss in an estrogen deficiency mice model and older women, although the magnitude of the effect was small. We hypothesized that long-term treatment with *L reuteri* could result in clinically relevant skeletal benefits in postmenopausal osteoporosis.

**Objective:**

To evaluate whether daily supplementation with *L reuteri* vs placebo could reduce early postmenopausal bone loss and whether the effects remained or increased over time during 2 years of treatment.

**Design, Setting, and Participants:**

A double-blind, randomized, placebo-controlled clinical trial was conducted between December 4, 2019, and October 6, 2022, at a single center in Gothenburg, southwestern Sweden. Participants were recruited by online advertisements, and letters were sent to 10 062 women aged 50 to 60 years. Responding women (n = 752) underwent telephone screening, resulting in 292 women being invited to a screening visit. Of those who were screened, 239 women met all inclusion criteria and had no exclusion criteria.

**Interventions:**

Capsules with *L reuteri* in 2 doses, 5 × 10^8^ (low dose) or 5 × 10^9^ (high dose) colony-forming units, taken twice daily or placebo were administered. All capsules also included cholecalciferol, 200 IU.

**Main Outcomes and Measures:**

The primary outcome was the relative change in tibia total volumetric bone mineral density (vBMD) over 2 years. Secondary outcomes included relative change in areal BMD of the lumbar spine and total hip, bone turnover markers C-terminal telopeptide cross-links of collagen type I and type I procollagen intact N-terminal propeptide, as well as tibia trabecular bone volume fraction and cortical vBMD. Both intention-to-treat and per-protocol analyses were conducted.

**Results:**

A total of 239 postmenopausal women (median age, 55 [IQR, 53-56] years) were included. Tibia vBMD (primary outcome), hip and spine vBMD, and tibia cortical area and BMD decreased significantly in all groups, with no group-to-group differences (percent change tibia vBMD high dose vs placebo least-squares means, −0.08 [95 CI, −0.85 to 0.69] and low dose vs placebo least-squares means, −0.22 [95% CI, −0.99 to 0.55]). There were no significant treatment effects on any other predefined outcomes. A prespecified sensitivity analysis found a significant interaction between body mass index (BMI) and treatment effect at 2 years. No significant adverse effects were observed.

**Conclusions and Relevance:**

In this randomized clinical trial of 239 early postmenopausal women, supplementation with *L reuteri* had no effect on bone loss or bone turnover over 2 years. The observed interaction between BMI and treatment effect warrants further investigation.

**Trial Registration:**

ClinicalTrials.gov Identifier: NCT04169789

## Key Points

**Question:**

Can daily supplementation with the probiotic *Limosilactobacillus reuteri* 6475 vs placebo reduce or prevent age-dependent bone loss in early postmenopausal women?

**Findings:**

In this randomized, placebo-controlled clinical trial with 239 early postmenopausal women, *L reuteri* 6475 supplementation vs placebo did not affect change in tibia volumetric bone mineral density (BMD). In addition, no effect on BMD of the lumbar spine or hip was observed over 24 months.

**Meaning:**

The findings of this trial suggest that *L reuteri* 6475 does not affect BMD of the tibia, spine, or hip in early postmenopausal women and should not be recommended to women at this age to prevent bone loss.

## Teaser

This randomized clinical trial examines the use of *Limosilactobacillus reuteri* supplementation in early postmenopausal women for the prevention of bone loss.

## Introduction

Osteoporosis, a disease characterized by low bone mineral density (BMD) and impaired bone microstructure leading to increased risk of fracture, represents a major public health concern with pronounced implications for morbidity and mortality.^1,2^ The total annual cost for osteoporotic fractures has been estimated to $20 billion in the US and $30 billion in the European Union.^3^ Osteoporosis treatments used today are only indicated for patients with already established low BMD, emphasizing the need for safe and effective novel treatments to prevent osteoporosis development.^4^

The gut microbiome has several important physiologic functions, including regulation of the immune system, protection against pathogen overgrowth, intestinal endocrine signaling, biosynthesis of vitamins, and contribution to energy biogenesis.^5^ Dysbiosis of the gut microbiota has been implicated in diseases such as obesity, diabetes, sarcopenia, osteoporosis, and autoimmune diseases.^5,6,7,8^

*Limosilactobacillus reuteri* ATCC PTA 6475 (*L reuteri*) is one of the few indigenous *Lactobacillus* species present in infants as well as adults.^9,10^ Clinical trials have extensively explored the probiotic and health-promoting effects of *L reuteri* in both adults and children.^11,12,13^ *Limosilactobacillus reuteri* protects against estrogen deficiency bone loss in mice through mechanisms that may involve reduced intestinal permeability and inflammation due to estrogen deficiency.^14,15,16,17^

A placebo-controlled randomized clinical trial (RCT) found that *L reuteri* reduced bone loss over 12 months in women aged 76 years by approximately half,^18^ but the magnitude of effect was limited. However, if the treatment effects increase over time, long-term treatment may result in clinically relevant differences in BMD, which are then likely to affect the risk of fracture in this population.^19^ In this RCT, we aimed to evaluate whether daily supplementation with *L reuteri* vs placebo could reduce early postmenopausal bone loss and whether the effects remained or increased during the 2-year study.

## Methods

### Study Design

**Figure 1.**
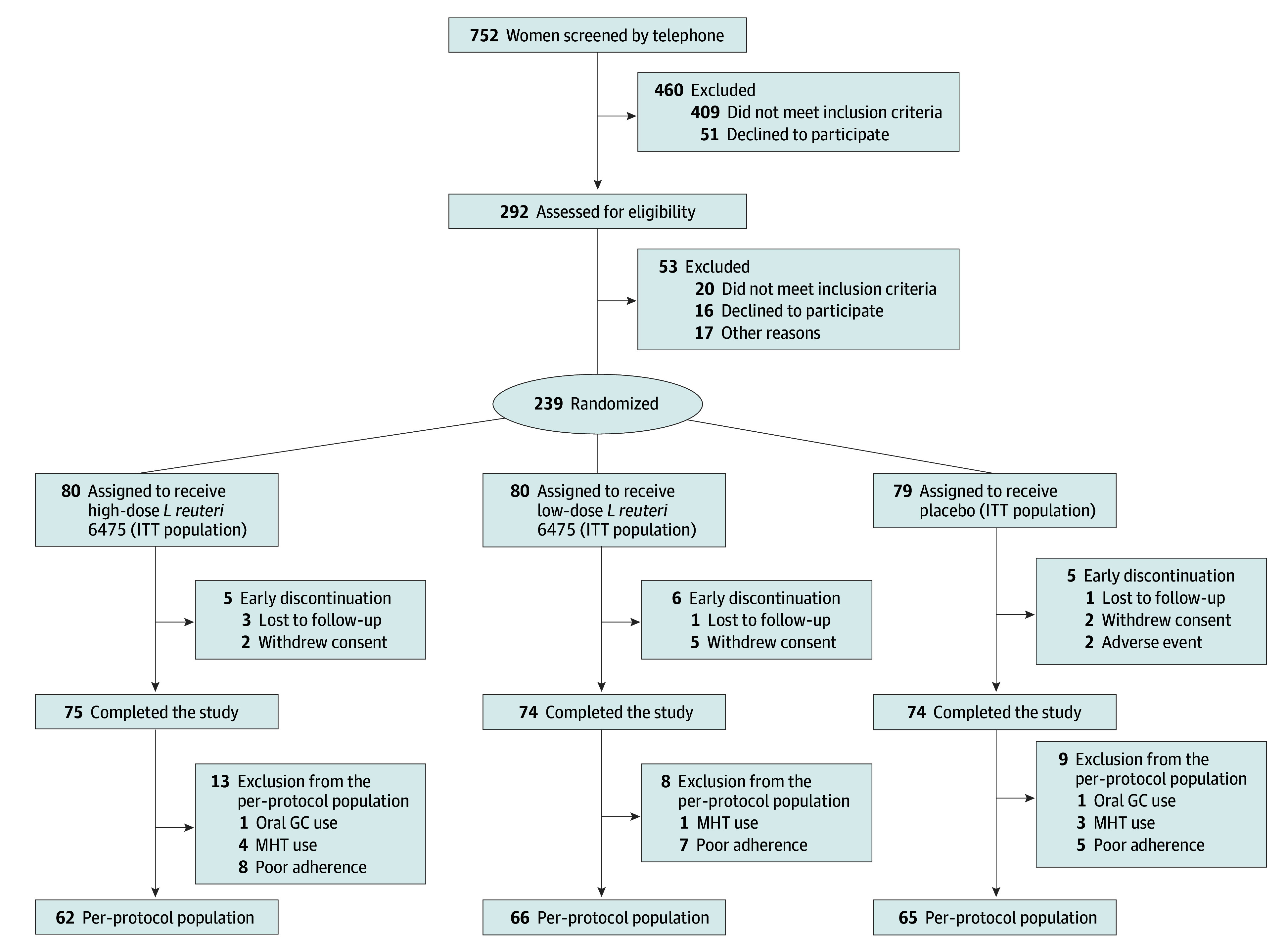
Participant Flowchart for *Limosilactobacillus reuteri* vs Placebo Groups GC indicates glucocorticoid; ITT, intention-to-treat; and MHT, menopausal hormone therapy.

The Early Postmenopausal Bone Loss with *Lactobacillus reuteri* II study is a double-blind, randomized, placebo-controlled, single-center RCT performed in the greater Gothenburg area in southwestern Sweden between December 4, 2019, and October 6, 2022. The exclusion criteria, inclusion criteria, study procedures, and predefined outcomes are available in Supplement 1. The study was approved by the Swedish Ethical Review Authority. The study is reported following the Consolidated Standards of Reporting Trials (CONSORT) reporting guideline for RCTs (Figure 1).

### Participants

Study participants were recruited by online advertisements and letters that were sent to 10 062 women aged 50 to 60 years. Women who contacted the clinic (n = 752) first underwent a telephone screening process, resulting in 292 women who were invited to a screening visit. Of those who were screened, 239 women met all inclusion criteria, had no exclusion criteria, and gave informed consent in writing and verbally to participate (Figure 1). Inclusion criteria were willingness to participate, availability throughout the study period, no menstruation within the past 1 to 4 years, and serum 25-OH-vitamin D levels greater than 10 ng/mL (to convert to nanomoles per liter, multiply by 2.496). Only randomized participants were reimbursed for travel and parking costs and received 900 Sk (US $82.62) after study completion. Exclusion criteria included: a T score less than −2.5 SDs for BMD combined with a 10-year probability of major osteoporotic fracture according to the fracture risk assessment tool (FRAX)^20^ of 20% or higher; severe osteoporosis, defined as a T score less than −3.0 SDs in either total hip, femoral neck, or lumbar spine; vertebral fracture diagnosed using lateral spine imaging with dual-energy x-ray absorptiometry (DXA); and previous (within the past 5 years) use of antiresorptive treatment, including systemic hormone therapy (estrogen), bisphosphonates, strontium ranelate, or denosumab. Further Study Protocol details are provided in Supplement 1.

### Randomization and Blinding

After inclusion, women were randomized to 1 of 3 treatment groups, with 2 groups receving different capsules with identical appearance of the active *L reuteri* treatment, high and low dose, and 1 group receiving placebo. The randomization visit took place within 4 weeks of the screening visit. The sponsor (BioGaia AB) carried out the randomization performed in blocks of 8 study participants generated using the website Randomization.com.^21^ The investigators had no access to the randomization code and remained blinded until study end, completion of the statistical analysis plan (Supplement 2), and database lock.

### Procedures

The study product consisted of capsules with *L reuteri* 6475 (BioGaia AB) in 2 different doses, 5 × 10^8^ (low does) or 5 × 10^9^ (high dose) colony-forming units mixed with maltodextrin powder, taken twice daily (1 in the morning: total dose, 1 × 10^9^; 1 in the evening: total dose, 1 × 10^10^), or placebo that contained maltodextrin powder only. All capsules also included cholecalciferol, 200 IU.

### Questionnaires

Standardized questionnaires regarding lifestyle habits as well as medical and drug history, risk factors for osteoporosis and fracture, Food Frequency Questionnaire,^22^ exercise habits (International Physical Activity Questionnaire),^23^ and gastrointestinal symptoms (Gastrointestinal Symptom Rating Scale [GSRS])^24^ were completed at baseline. Participants attended quarterly visits to report adherence and possible adverse events, return any remaining study product and receive new study product, and provide blood, serum, and feces samples.

### Bone Measurements

Bone mineral density of the total hip and lumbar spine and body composition of the total body were measured at baseline, 1 year, and 2 years using scan imaging (GE Lunar iDXA; GE Lunar). The coefficients of variation for BMD measurements, assessed in 8 men and 24 women, were 0.48% for the total hip, 0.77% for the femoral neck, and 0.73% for the lumbar spine. Lateral spine imaging was used to diagnose and classify vertebral compression fractures.^25^

Volumetric BMD (vBMD) and bone microstructure parameters were measured at baseline, after 1 year, and after 2 years in the distal tibia using high-resolution peripheral quantitative computed tomography (XtremeCT; Scanco Medical AG) using a protocol described previously.^26,27^ After processing the images,^28^ the following variables were obtained: total vBMD (milligrams per cubic centimeter), cortical thickness (millimeters), cortical vBMD, and trabecular bone volume fraction (percent). The coefficients of variation at our unit, assessed in 30 women aged 75 to 80 years, were 0.2% (total vBMD), 0.5% cortical thickness, 0.3% (cortical vBMD), and 0.5% (trabecular bone volume fraction).

### Blood Biochemistry

Fasting morning blood samples were collected from all study participants. Aliquots of serum and plasma were stored at –80 °C until analysis. Serum type I procollagen intact N-terminal propeptide (PINP), C-terminal telopeptide cross-links of collagen type I (CTX), 25-OH-vitamin D and calcium, and plasma short-chain fatty acids butyrate, acetate, and propionate were analyzed (eMethods in Supplement 3).

### Outcomes

The primary outcome was the relative change in tibia total vBMD after 2 years of treatment compared with placebo. Effects of treatment vs placebo were also investigated after 1 year and 2 years for the following secondary outcomes: BMD at the total hip and lumbar spine; tibia trabecular bone volum fraction; tibia cortical area; tibia cortical vBMD; tibia total vBMD (12 months); CTX and PINP; and plasma butyrate, acetate, and propionate.

### Statistical Analysis

The study had the power of more than 99% to detect differences between groups based on Bonferroni-Holm correction, an anticipated dropout rate of 15% at 2 years, and a presumed mean (SD) decrease in tibia total vBMD of −3.50% (1.6%) in the placebo group vs 1.75% (1.6%) in the high-dose group or −2.10% (1.6%) in the low-dose group. A statistical analysis plan was developed, dated, and signed by the principal investigator (M.L.) and study statistician (A.P.) before unblinding (Supplement 2). The primary and all secondary variables were analyzed both for the intention-to-treat (ITT) population (all randomized participants) and per-protocol (PP) population (those who completed the study and were not in violation of the exclusion criteria). The PP population excluded women who during the study used osteoporosis medication, menopausal hormone therapy (protocol amendment: December 1, 2022, as described) and described in the statistical analysis plan), oral glucocorticoids for more than 2 weeks, or other probiotic supplements. All tests were 2-tailed. To account for type 1 error, a preplanned α level of .025 was used for the 2 active comparisons against the placebo with respect to the primary end point. Bonferroni-Holm adjustment was planned to be applied to the secondary end points.

Additional exploratory subgroup analyses that were not predefined and described in the statistical analysis plan (Supplement 2) were performed using logistic regression, to investigate which baseline variables were associated with a positive treatment response (defined as a percent change in total vBMD at 2 years of more than −1.0). The relation between 2 continuous variables was described and tested by the Pearson correlation coefficient in the case of normally distributed data and otherwise by the Spearman correlation coefficient.

## Results

A total of 239 early postmenopausal women (median age, 55 [IQR, 53-56] years) were randomly assigned to receive high-dose *L reuteri* (n = 80), low-dose *L reuteri* (n = 80), or placebo (n = 79) (Figure 1). The ITT analysis included 223 women; PP analysis, 193; and safety analysis, 239 (eTable 1 in Supplement 3). In total, 223 participants (93.3%) completed the study (Figure 1). At randomization, the groups were in general well balanced in terms of anthropometrics, medical history, gastrointestinal symptoms (according to the GSRS), dietary intake, physical activity, bone characteristics (DXA-derived BMD at the spine and hip and high-resolution peripheral quantitative computed tomography–derived vBMD, bone geometry, and bone microstructure at the tibia), vitamin D levels, bone turnover markers CTX and PINP, and 10-year fracture probability according to FRAX (Table 1; eTable 2 and eTable 3 in Supplement 3).

**Table 1.** Demographic and Baseline Characteristics of the Intention-to-Treat Population

Characteristic	*Limosilactobacillus reuteri*		Placebo (n = 79)
	High dose (n = 80)	Low dose (n = 80)	
Age, median (IQR), y	55 (52 to 56)	55 (53 to 56)	55 (53 to 56)
Height, mean (SD), cm	166.9 (6.4)	166.0 (6.6)	168.0 (7.8)
Weight, median (IQR), kg	66.5 (61.9 to 77.5)	65.2 (60.3 to 76.5)	68.8 (59.3 to 79.8)
BMI, median (IQR)	23.9 (22.1 to 27.5)	24.5 (21.9 to 27.9)	23.7 (21.4 to 28.3)
Total body fat, median (IQR), %	37.5 (31.0 to 41.8)	36.0 (30.5 to 42.9)	35.5 (31.0 to 42.9)
Appendicular LMI, median (IQR), kg/m^2^	6.5 (6.0 to 7.0)	6.7 (6.1 to 7.1)	6.6 (6.3 to 7.0)
Current smoking, No. (%)	2 (2.5)	2 (2.5)	5 (6.3)
High alcohol consumption, No. (%)^a^	0	0	0
Parental hip fracture, No. (%)	8 (10.0)	12 (15.0)	11 (14.1)
Run-in period, median (IQR), d	14.0 (9.5 to 20.0)	14.0 (10.0 to 18.5)	13.0 (8.0 to 17.0)
Previous glucocorticoid use, No. (%)	0	3 (3.8)	2 (2.5)
Serum 25-OH-vitamin D, median (IQR), ng/mL	31.0 (23.0 to 36.9)	30.2 (24.6 to 34.5)	28.8 (24.4 to 35.7)
Plasma total calcium, mean (SD), mg/dL	9.44 (0.28)	9.36 (0.24)	9.48 (0.24)
PINP, median (IQR), μg/L	62.3 (47.4 to 86.7)	77.9 (58.2 to 100.9)	79.8 (61.7 to 105.6)
CTX, median (IQR), ng/mL	0.3 (0.2 to 0.5)	0.4 (0.3 to 0.4)	0.3 (0.3 to 0.5)
GSRS total score, median (IQR)^b^	1.3 (1.1 to 1.7)	1.3 (1.1 to 1.6)	1.3 (1.1 to 1.5)
Energy intake, median (IQR), kcal/d	1569 (1241 to 1948)	1831 (1563 to 2331)	1748 (1415 to 2201)
Protein intake, median (IQR), g/d	67 (54 to 86)	76 (59 to 91)	73 (61 to 98)
Fat intake, median (IQR), g/d	65 (52 to 86)	76 (58 to 100)	75 (55 to 96)
Carbohydrate intake, median (IQR), g/d	157 (120 to 196)	191 (138 to 254)	173 (124 to 216)
Fiber intake, median (IQR), g/d	21 (15 to 30)	26 (19 to 35)	24 (16 to 33)
Salt intake, median (IQR), g/d	5.2 (4.2 to 6.6)	5.7 (5.1 to 7.4)	5.6 (4.7 to 7.7)
Calcium intake, median (IQR), mg/d	992 (861 to 1341)	1177 (900 to 1537)	1182 (894 to 1483)
Physical activity, median (IQR), MET/wk	2522 (1378 to 4804)	2650 (1499 to 4515)	2991 (1725 to 5349)
Tibia cortical area, mean (SD), mm	117.6 (20.6)	115.4 (21.1)	110.8 (19.8)
Tibia total vBMD, median (IQR), mg/cm^3^	292.1 (47.0)	283.2 (44.5)	283.4 (48.3)
Tibia cortical vBMD, median (IQR), mg/cm^3^	854.3 (57.5)	843.8 (52.0)	842.1 (52.4)
Tibia trabecular BV/TV, median (IQR), %	0.13 (0.03)	0.13 (0.03)	0.13 (0.03)
BMD total hip, median (IQR), g/cm^2^	0.97 (0.12)	0.96 (0.10)	0.97 (0.12)
BMD femoral neck, median (IQR), g/cm^2^	0.93 (0.11)	0.92 (0.10)	0.93 (0.11)
BMD lumbar spine, median (IQR), g/cm^2^	1.15 (0.14)	1.13 (0.15)	1.15 (0.16)
T score total hip (SD)	−0.31 (0.93)	−0.36 (0.77)	−0.30 (0.93)
T score lumbar spine (SD)	−0.53 (−1.28 to 0.33)	−0.57 (−1.60 to 0.25)	−0.75 (−1.27 to 0.33)
FRAX MOF (with BMD)	5.20 (4.52 to 7.22)	6.17 (5.00 to 9.11)	5.30 (4.38 to 8.61)
FRAX MOF (without BMD)	5.44 (4.84 to 6.52)	6.03 (4.88 to 10.32)	5.61 (4.80 to 8.31)
FRAX hip fracture (with BMD)	0.40 (0.23 to 0.78)	0.55 (0.28 to 0.97)	0.50 (0.18 to 0.94)
FRAX hip fracture (without BMD)	0.75 (0.57 to 1.06)	0.99 (0.60 to 1.62)	0.79 (0.54 to 1.29)
Prevalent falls, No. (%)	15 (18.8)	14 (17.5)	17 (21.5)
Prevalent fracture, No. (%)	9 (11.3)	21 (26.3)	11 (13.9)
Years since menopause, median (IQR)	2.0 (1.0 to 3.0)	2.0 (1.0 to 3.0)	2.0 (2.0 to 3.0)
Calcium and vitamin D supplement use 3 mo before baseline, No. (%)^c^	6 (7.6)	4 (5.1)	3 (3.8)

Abbreviations: BMD, bone mineral density; BMI, body mass index (calculated as weight in kilograms divided by height in meters squared); BV/TV, bone volume fraction; CTX, C-terminal telopeptide cross-links of collagen type I; FRAX, fracture risk assessment tool; GSRS, Gastrointestinal Symptom Rating Scale; LMI, lean mass index; MET, metabolic equivalent of task; MOF, major osteoporotic fracture; PINP, procollagen intact N-terminal propeptide; vBMD, volumetric BMD.

SI conversion factors: To convert calcium to milligrams per deciliter, multiply by 0.25; 25-OH-vitamin D to nanomoles per liter, multiply by 2.496.

^a^ High alcohol consumption = 3 or more standard units/d.

^b^ High dose, n = 79; low dose, n = 80; placebo, n = 78.

^c^ High dose, n = 79; low dose, n = 79; placebo, n = 79.

In the ITT population, tibia total vBMD, lumbar spine BMD, total hip BMD, tibia cortical area, and tibia cortical vBMD decreased significantly in all 3 investigated groups at both 1 and 2 years. There were no group-to-group differences in percent change in tibia vBMD high dose vs placebo (least-squares mean, −0.08 [95% CI, −0.85 to 0.69]) and low dose vs placebo (least-square mean, −0.22 [95% CI, −0.99 to 0.55]). There were no significant treatment effects on any other predefined bone outcomes (Table 2). A significantly higher percent change in PINP was observed at 1 year for the high-dose group compared with placebo and at 2 years for both the high- and low-dose groups compared with placebo. Following adjustment for multiple comparisons, these differences were not significant. Multiple imputation was used to allow efficacy analysis and group-to-group comparison between the change in tibia total vBMD in all randomized participants (n = 239). In this analysis, no significant difference was found between either *L reuteri* dose and placebo (eTable 4 in Supplement 3). Similarly, no group-to-group differences were found for any of the studied bone characteristics (primary and secondary) at year 1 or at year 2 in the PP population following adjustment for multiple comparisons (eTable 5 in Supplement 3). Overall, mean (SD) adherence to the study product was high, ranging from 87.5% (24.6%) in the high-dose *L reuteri* group to 93.6% (12.9%) in the placebo group (eTable 6 in Supplement 3).

**Table 2.** Analysis of the Relative Change in Bone Characteristics and Bone Turnover Markers of the Intention-to-Treat Population^a^

Percent change (%)	Visit	*Limosilactobacillus reuteri*				Placebo		Difference			
		High dose		Low dose				High dose – placebo		Low dose – placebo	
		LS means (95% CI)	*P* value	LS means (95% CI)	*P* value	LS means (95% CI)	*P* value	LS means (95% CI)	*P* value	LS means (95% CI)	*P* value
Tibia total volumetric BMD	Year 1	−1.21 (−1.65 to −0.77)	<.001	−1.02 (−1.46 to −0.59)	<.001	−1.19 (−1.62 to −0.76)	<.001	−0.02 (−0.63 to 0.60)	.96	0.17 (−0.45 to 0.78)	.59
	Year 2	−2.39 (−2.93 to −1.84)	<.001	−2.53 (−3.07 to −1.98)	<.001	−2.31 (−2.85 to −1.76)	<.001	−0.08 (−0.85 to 0.69)	.84	−0.22 (−0.99 to 0.55)	.57
BMD lumbar spine	Year 1	−1.29 (−1.85 to −0.72)	<.001	−1.81 (−2.37 to −1.25)	<.001	−1.09 (−1.65 to −0.53)	<.001	−0.20 (−0.99 to 0.60)	.63	−0.72 (−1.51 to 0.08)	.08
	Year 2	−1.95 (−2.62 to −1.28)	<.001	−2.36 (−3.03 to −1.68)	<.001	−1.88 (−2.56 to −1.21)	<.001	−0.07 (−1.02 to 0.88)	.89	−0.47 (−1.43 to 0.48)	.33
BMD total hip	Year 1	−1.55 (−2.04 to −1.07)	<.001	−1.47 (−1.96 to −0.99)	<.001	−1.26 (−1.74 to −0.79)	<.001	−0.29 (−0.97 to 0.38)	.40	−0.21 (−0.89 to 0.46)	.53
	Year 2	−2.43 (−3.01 to −1.86)	<.001	−2.39 (−2.97 to −1.82)	<.001	−2.18 (−2.75 to −1.61)	<.001	−0.26 (−1.06 to 0.55)	.54	−0.22 (−1.03 to 0.59)	.60
Tibia trabecular bone volume fraction	Year 1	−0.15 (−0.64 to 0.35)	.56	−0.18 (−0.68 to 0.32)	.47	−0.46 (−0.94 to 0.03)	.07	0.31 (−0.38 to 1.00)	.38	0.28 (−0.42 to 0.97)	.44
	Year 2	−0.73 (−1.32 to −0.13)	.02	−1.20 (−1.80 to −0.61)	<.001	−1.25 (−1.84 to −0.66)	<.001	0.52 (−0.31 to 1.36)	.22	0.05 (−0.79 to 0.89)	.91
Tibia cortical area	Year 1	−2.19 (−2.86 to −1.52)	<.001	−1.92 (−2.59 to −1.25)	<.001	−2.00 (−2.67 to −1.33)	<.001	−0.19 (−1.14 to 0.76)	.69	0.08 (−0.87 to 1.02)	.87
	Year 2	−3.64 (−4.52 to −2.76)	<.001	−3.82 (−4.70 to −2.94)	<.001	−3.18 (−4.06 to −2.30)	<.001	−0.46 (−1.70 to 0.79)	.47	−0.64 (−1.88 to 0.61)	.32
Cortical volumetric BMD	Year 1	−0.94 (−1.20 to −0.67)	<.001	−0.71 (−0.97 to −0.45)	<.001	−0.78 (−1.03 to −0.52)	<.001	−0.16 (−0.53 to 0.21)	.40	0.07 (−0.30 to 0.43)	.72
	Year 2	−1.87 (−2.20 to −1.53)	<.001	−1.63 (−1.96 to −1.29)	<.001	−1.51 (−1.85 to −1.18)	<.001	−0.35 (−0.83 to 0.12)	.15	−0.11 (−0.59 to 0.36)	.64
CTX	Year 1	10.91 (1.38 to 20.44)	.02	11.58 (2.35 to 20.82)	.01	0.86 (−8.17 to 9.89)	.85	10.05 (−3.08 to 23.19)	.13	10.72 (−2.20 to 23.63)	.10
	Year 2	−0.94 (−10.34 to 8.45)	.84	8.15 (−1.16 to 17.47)	.09	2.98 (−6.18 to 12.13)	.52	−3.92 (−17.05 to 9.20)	.56	5.17 (−7.89 to 18.23)	.44
PINP	Year 1	5.58 (−1.52 to 12.68)	.12	0.57 (−6.56 to 7.69)	.88	−7.89 (−14.87 to −0.92)	.03	13.48 (3.50 to 23.45)	.008	8.46 (−1.50 to 18.42)	.10
	Year 2	0.57 (−8.50 to 9.64)	.90	0.40 (−8.76 to 9.56)	.93	−13.84 (−22.89 to −4.79)	.003	14.41 (1.58 to 27.24)	.03	14.24 (1.38 to 27.11)	.03

Abbreviations: BMD, bone mineral density; CTX, C-terminal telopeptide cross-links of collagen type I; LS, least-squares; PINP, procollagen intact N-terminal propeptide.

^a^ Mixed models for repeated measures were applied with percent difference as the outcome variable, visit, treatment group, interaction visit × treatment group as the main fixed effects; and baseline value as a covariate. An unstructured covariance pattern is used for correlated data repeated over time. Diagnostic plots of residuals were investigated and found satisfactory. No between-group differences were significant following Bonferroni-Holm adjustment.

During the study duration, there were no significant differences between the groups in any adverse event, in any severe adverse event, or in an adverse event leading to discontinuation of the study product. A higher proportion of participants reported any treatment-related adverse event in the placebo group than in the low-dose *L reuteri* group. There were no group-to-group differences in adverse events leading to discontinuation of the study product (Table 3). A detailed description of the distribution of adverse events and serious adverse events per treatment group is presented in eTable 7 in Supplement 3. The change in gastrointestinal symptoms was also studied using the GSRS scale. There were no significant differences between treatment groups in the change in any of the GSRS subdomains (reflux, abdominal pain, indigestion, diarrhea, and constipation) or in the total GSRS score (eTable 8 in Supplement 3).

**Table 3.** Summary of AEs of the Safety Population^a^

Variable	*Limosilactobacillus reuteri*, No. (%)		Placebo (n = 79)
	High dose (n = 80)	Low dose (n = 80)	
Any AE	80 (100)	74 (92.5)	77 (97.5)
Any SAE	1 (1.3)	4 (5.0)	6 (7.6)
Any treatment-related AE	46 (57.5)	36 (45.0)	51 (64.6)
Any treatment-related SAE	0	0	0
Any AE leading to discontinuation of study product	6 (7.5)	2 (2.5)	4 (5.1)
Any fracture	3 (3.8)	3 (3.8)	5 (6.3)

Abbreviations: AE, adverse event; SAE, severe adverse event.

^a^ For test between 2 groups with respect to dichotomous variables, the Fisher exact test was used.

In an exploratory but predefined analysis, the group-to-group changes in serum levels of the short-chain free fatty acids acetic acid, propionic acid, and butyric acid were investigated. Over 2 years of treatment, serum acetic acid levels decreased significantly more in the high-dose *L reuteri* group than in the placebo group. No other group-to-group differences in the change in short-chain fatty acids were observed (eTable 9 in Supplement 3).

**Figure 2.**
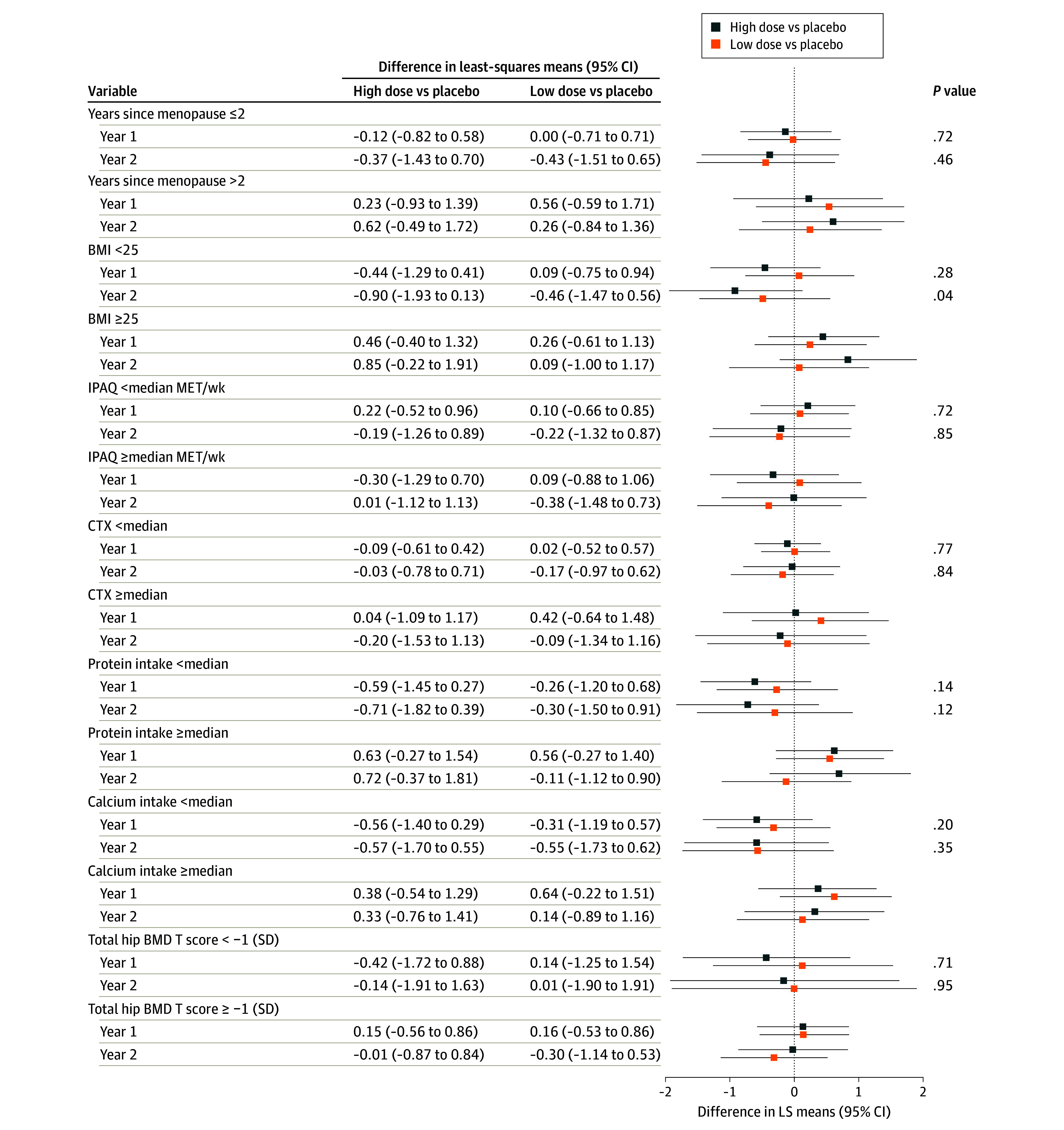
Subgroup Analyses of the Primary Efficacy Variable in the Intention-to-Treat Population Percent change in tibia ultradistal (standard site) total volumetric BMD (primary efficacy variable) is shown for subgroups of different baseline characteristics. Mixed models for repeated measures are applied with percent difference as the outcome variable; visit, treatment group, and interaction visit × treatment group as main fixed effects; and baseline value as covariate. An unstructured covariance pattern is used for correlated data repeated over time. BMD indicates bone mineral density; BMI, body mass index (calculated as weight in kilograms divided by height in meters squared); CTX, C-terminal telopeptide cross-links of collagen type I; IPAQ, International Physical Activity Questionnaire; and MET, metabolic equivalent of task.

A predefined subgroup analysis was conducted evaluating the relative change in tibia total vBMD in the ITT population for subgroups defined by years since menopause (<median years, ≥median years), body mass index (BMI) (<25, ≥25 [calculated as weight in kilograms divided by height in meters squared]), and International Physical Activity Questionnaire (<median metabolic equivalent of task [MET]/week,≥median MET/week). There were no significant differences in change in tibia total vBMD during the 2-year treatment between any of the *L reuteri* treatment groups and placebo for any subgroup (Figure 2; eTable 10 in Supplement 3). However, a significant interaction between BMI group and treatment group (*P* = .04) was observed for change at year 2 but not at year 1 (*P* = .28). A significant correlation between BMI and relative percent change in tibia total vBMD was observed both in the high-dose *L reuteri* (*r* = 0.38; *P* < .001) and in the low-dose *L reuteri* (*r* = 0.29; *P* = .01) groups but not in the placebo group (*r* = 0.15; *P* = .22) at 2 years (eFigure in Supplement 3).

Exploratory but not predefined analyses of treatment effect on percent change in tibia vBMD according to subgroups of CTX, protein intake, calcium intake, and low total hip BMD T score were also performed and did not reveal any interactions between these variables and treatment effect (Figure 2; eTable 10 in Supplement 3). Another exploratory analysis to identify differences in baseline traits between responders and nonresponders (defined as percent change in total tibia vBMD at 2 years of more than −1.0) was performed on all women in the high- and low-dose groups (eTable 11 in Supplement 3). Higher weight, BMI, total body fat percent, and 10-year fracture probability (FRAX) for major osteoporotic fracture (with and without BMD) were associated with a more positive treatment response.

## Discussion

In this RCT, supplementation with *L reuteri* for 24 months resulted in no significant differences in the primary outcome, the change in tibia total vBMD, or in any of the secondary outcomes compared with placebo. The lack of treatment effect on bone parameters was evident both in the ITT and PP populations, demonstrating that protocol violations did not mask any treatment effect of the study product. Similarly, multiple imputation was used to perform analyses of efficacy and group-to-group comparisons with all participants in the ITT population, and this analysis showed no significant differences in the primary outcome variable between either *L reuteri* dose vs the placebo group.

The 3 randomization groups were in general well matched across multiple parameters, encompassing anthropometric measures, medical history, gastrointestinal symptoms, 10-year fracture probabilities, physical activity, and bone characteristics, including BMD at the spine and hip and the primary outcome variable, vBMD at the tibia. However, an imbalance in calcium and protein intake was observed between the high-dose group and placebo group, with the latter having greater intakes of both of these nutrients. This may have affected the longitudinal changes in bone parameters since it is well established that increased calcium intake as well as protein intake both result in increased BMD.^29,30^ Arguing against this hypothesis, additional exploratory analysis of treatment effect according to calcium and protein intake was performed and did not reveal any association between these baseline parameters and treatment effect on percent change in tibia vBMD over 2 years.

A predefined subgroup analysis examined the relative change in tibia total vBMD in the ITT population, stratifying participants based on years since menopause, BMI, and physical activity levels. The analysis revealed no substantial differences in changes in tibia total vBMD over the 2-year treatment period between any of the *L reuteri* treatment groups and placebo, regardless of the subgroup except the BMI subgroups. A significant interaction was observed between BMI groups and treatment, with a more favorable treatment response in participants with high vs low BMI. Furthermore, a statistically significant correlation between BMI and the relative change in tibia total vBMD was observed in both the high- and low-dose *L reuteri* groups at year 2, but such a correlation was not observed in the placebo group, indicating that *L reuteri* treatment may have a beneficial effect on bone loss in those with high BMI. It is plausible that *L reuteri* has positive effects on bone metabolism if dysbiosis is present, which is more common in obesity^31^ and with advanced age. Thus, the previously observed reduction in bone loss with *L reuteri* in older women^18^ and the herein observed difference in effect in favor of those with high BMI could be due to dysbiosis in both of these populations.

Additional analyses that were not predefined using logistic regression to identify baseline parameters associated with a positive treatment response, defined as a percent change greater than −1 over 2 years, revealed that higher weight, BMI, total body fat, and 10-year fracture probability (FRAX) for major osteoporotic fracture, both with and without including BMD, were associated with more positive treatment response, which indicates that *L reuteri* supplementation may be more effective in a population with higher fracture risk and higher prevalence of obesity. *Limosilactobacillus reuteri* did not increase gastrointestinal symptoms or cause more adverse events than the placebo treatment, further supporting the well-documented safety of this probiotic supplementation.^12,13^

Several experimental studies support the notion that the gut microbiota and specific *Lactobacillus* strains have a role in estrogen deficiency–induced bone loss.^14,16,32^ It was shown that estrogen deficiency caused by ovariectomy does not result in bone loss in germ-free mice.^16^ Another study investigating mice with present gut microbiota found that ovariectomy-induced estrogen deficiency leads to increased intestinal permeability, upregulated osteoclastogenic cytokines such as receptor activator of nuclear factor κ-β ligand and interleukin-17, resulting in trabecular bone loss that could be prevented by supplementation with the probiotic *Lactobacillus rhamnosus*. Furthermore, *L reuteri* protected against ovariectomy-induced bone loss through attenuating osteoclasts by suppressing ovariectomy-induced bone marrow CD4^+^ T lymphocytes.^14^ Additional attributes of *L reuteri* encompass robust resilience within acidic environments and the unique capability to modulate inflammatory cascades in human macrophages, notably by suppressing tumor necrosis factor–mediated inflammation.^33^ The probiotic *L rhamnosus* further unveiled its potential in augmenting bone mass by increasing serum levels of butyrate, with downstream effects on regulatory T cells and bone anabolic ligands, such as Wnt10b.^34^ *Limosilactobacillus reuteri* was able to reduce the suppression of Wnt10b in type 1 diabetic murine models,^35^ suggesting potential roles in modifying intestinal inflammation and influencing the production of bone anabolic ligands, but in the present study, we did not observe any effect of *L reuteri* treatment on circulating levels of butyric acid.

Few well-powered, high-quality RCTs have investigated the effect of different probiotics on bone parameters in postmenopausal women.^36^ A meta-analysis suggested that supplementation with some probiotic strains may have positive effects on bone health.^37^ For example, it was reported that a mix of 3 *Lactobacillus* strains (*L paracasei* 8700:2 [*DSM* 13434], *L plantarum* Heal 9 [*DSM* 15312], and *L plantarum* Heal 19 [*DSM* 15313]) protects against lumbar spine bone loss in healthy early postmenopausal women.^36^ In a previous RCT involving 90 older women, supplementation with *L reuteri* for 12 months reduced loss of tibia total vBMD by half compared with the placebo group.^18^ The discrepancy from the present trial results could be attributable to the fact that the treatment is only effective if BMD is low or the population is older, a condition associated with dysbiosis.^38^

### Strengths and Limitations

This study possesses several notable strengths. First, it adopted an RCT design, which is considered the standard in clinical research, ensuring a rigorous evaluation of the intervention’s effects. The study maintained a large sample size with a low dropout rate (93.3% completed the study; thus, 6.7% dropped out vs 15% expected), which enhanced the statistical power and generalizability of the findings. Adherence to the study product was high in all groups, with only minor variations between the *L reuteri* dose groups and the placebo group. Furthermore, the comprehensive assessment of bone health included a wide array of parameters, such as BMD, bone geometry and bone microstructure, and bone turnover markers, providing a thorough examination of the intervention’s effect on bone health. Moreover, the unprecedented study duration offers insights into short-term effects and potential longer-term effects of *L reuteri* supplementation on bone health.

The study also has limitations. The study population consisted of early postmenopausal women, limiting the generalizability of the findings to other demographic groups. Variations in dietary protein intake and health parameters between treatment groups at baseline may introduce potential confounding factors. Since the *L reuteri* treatment did not achieve the primary objective, the hypothesis was rejected, and the testing sequence for significance adjustments using Bonferroni-Holm failed to detect any confirmed significant effects of treatment on any investigated outcome, primary or secondary. Thus, all reported significant results should be interpreted as exploratory and not confirmatory. The lack of effect could be due to an insufficient dose or viability of *L reuteri*, but a previous RCT^18^ found an effect on bone loss using the high dose used in the present study, and the producer of *L reuteri* (BioGaia AB) performed frequent viability checks to ensure the viability of the strain for the whole study duration. In addition, while the interaction between BMI and treatment group at 2 years is intriguing, its significance requires further investigation to elucidate its underlying mechanisms and clinical implications.

## Conclusions

This large and well-powered RCT did not demonstrate any significant effect of high- or low-dose *L reuteri* on bone health in early postmenopausal women over 2 years. A prespecified sensitivity analysis found an interaction between BMI and treatment effect, which warrants further investigation.

## Notes

Supplement 1. **Trial Protocol and Amendmen**t

Supplement 2.
Statistical Analysis Plan

Supplement 3. **eMethods**. Extended Methods - Blood Biochemistry Analysis and Statistical Methods
eReference
**eTable 1.** Patient Disposition and Data Sets Analyzed (ITT Population) **eTable 2.** Medical History (ITT Population) **eTable 3.** Prior Medications (ITT Population) **eTable 4.** Change in the Tibia Total Volumetric BMD According to Treatment Using Multiple Imputation in the ITT Population **eTable 5.** Main and Sensitivity Analyses of the Percent Change in Bone Characteristics and Bone Turnover Markers in the PP Population **eTable 6.** Compliance (Safety Population) **eTable 7.** Adverse Events by System Organ Class and per Treatment in the Safety Population **eTable 8.** Analyses of the GSRS in the Safety Population **eTable 9.** Exploratory Analyses of the Change in Serum Short-Chain Fatty Acids in the ITT Population **eTable 10.** Subgroup Analyses of the Primary Efficacy Variable (ITT Population) **eTable 11.** Logistic Regression for Responders Based on the Responders Defined as Percent Change in Total Tibia vBMD at Two Years>-1.0 (All High- and Low-Dose Participants Included) **eFigure.** Pearson Correlation Between BMI and % Change in Tibia Total vBMD by Treatment Group Over Time (ITT Population)

Supplement 4.
Data Sharing Statement
