# Supplementary material for: Limosilactobacillus reuteri 6475 and Prevention of Early Postmenopausal Bone Loss: A Randomized Clinical Trial
Source: JAMA Netw Open. 2024 Jun 12;7(6):e2415455. doi: 10.1001/jamanetworkopen.2024.15455 (PMC11170297; doi:10.1001/jamanetworkopen.2024.15455)
Supplement: Supplement 1. — Trial Protocol and Amendment [file jamanetwopen-e2415455-s001.pdf]

Prevention of Early Postmenopausal Bone Loss with *Lactobacillus reuteri* – A Randomized, Placebo-Controlled, Single Centre Clinical Trial

DATE OF PROTOCOL

June 28th, 2019

STUDY PRODUCT

L. reuteri ATCC PTA 6475

PRINCIPAL INVESTIGATOR

Mattias Lorentzon, MD, Professor

CO-INVESTIGATORS

Anna Nilsson, MD, Associate Professor

Daniel Sundh, PhD

Fredrik Bäckhed, Professor

Mattias Lorentzon, principal investigator

Gothenburg, June 28h, 2019

Table of Contents

INTRODUCTION AND RATIONALE *3*
Research hypothesis 3
Study rationale 3
INVESTIGATIONAL PLAN AND PROCEDURES *5*
Study design 5
Objectives 7
SUBJECTS AND METHODS *7*
Study Product and Dosage 7
Intervention in normal diet 8
Eligibility 8
Analyses 9
Adherence 12
Ethical review and informed consent 12
Study period 12
Tabulated flow scheme for the study .... Error! Bookmark not defined.
Monitoring 14
Case Report Forms 14
Changes to the Study Protocol 14
Data analysis 14
Statistical analyses 14
Final Study Report 15
Indemnity 16
SAFETY CONSIDERATIONS *16*
Scientific background 16
Expected safety profile of *L. reuteri* ATCC PTA 6475 19
Other safety considerations 20
Adverse Events 20
Serious Adverse Events 21
Reporting Serious Adverse Events 21
References 21

Prevention of Early Postmenopausal Bone Loss with *Lactobacillus reuteri* – A Randomized, Placebo-Controlled, Single Centre Clinical Trial

INTRODUCTION AND RATIONALE

Research hypothesis

The aim of this planned randomized, double-blind, placebo-controlled trial is to investigate if daily supplementation with *Lactobacillus reuteri* ATCC PTA 6475 (*L. reuteri* 6475) can reduce early postmenopausal bone loss after two years of treatment.

Study rationale

Osteoporosis is a disease characterized by bone fragility resulting in bone fractures, which lead to increased morbidity and mortality. Women in Sweden have among the highest risks of hip and vertebral fractures in the world (1). Every second woman and every fifth man will sustain a fragility fracture during their lifetime in Sweden. Following menopause, bone resorption exceeds bone formation, leading to net bone loss and bone fragility. Loss of sex steroids and inflammation contribute to this bone loss. Patients with rheumatic conditions have greater bone loss and are at increased risk of sustaining fractures (2). Vitamin D is important for calcium uptake and bone mineralization. Vitamin D deficiency is known to cause osteomalacia and increased fracture risk(3). Lactobacilli are commensal bacteria common to the gut of all mammals studied and lactobacilli are considered to be safe for human consumption worldwide (4, 5). *L. reuteri* is a species of lactobacillus that naturally inhabits the gastrointestinal tract of humans and is one of the few autochthonous (indigenous) *Lactobacillus* species in infants as well as adults (6). *L. reuteri* has been widely studied in clinical trials and has been shown to have probiotic, health-promoting effects in both adults and children (7). *L. reuteri* has been shown in numerous studies

to be safe for human consumption and it has been shown to colonise the human gastrointestinal tract (8).

The 6475 L. reuteri strain has a typical sugar fermentation pattern, reuterin production and growth characteristic. Identification using 16SrRNA gene analysis has revealed that it has 99% similarity with the type strain of *L. reuteri* thus confirming that it belongs to this well-known and well-studied species that is considered safe for human consumption. In a mouse model, micro-computed tomography demonstrated that *L. reuteri* 6475 treatment increased male trabecular bone parameters (mineral density, bone volume fraction, trabecular number, and trabecular thickness) in the distal femur metaphyseal region as well as in the lumbar vertebrae. Cortical bone parameters were not significantly affected. Dynamic and static histomorphometry and serum re-modelling parameters indicated that *L. reuteri* ingestion increases osteoblast serum markers and dynamic measures of bone formation in male mice. However, no effects were seen in female mice in this model (9). In contrast, in an ovariectomized (Ovx) mouse menopausal model, *L. reuteri* 6475 treatment significantly protected Ovx mice from bone loss. Osteoclast bone resorption markers and activators (Trap5 and RANKL) as well as osteoclastogenesis were significantly decreased in *L. reuteri* 6475 treated mice. Consistent with this, *L. reuteri* 6475 suppressed Ovx-induced increases in bone marrow CD4+ T-lymphocytes (which promote osteoclastogenesis) and directly suppressed osteoclastogenesis in vitro (10). In vitro studies indicate that this strain has strong tolerance to acid environments, as do many other *L. reuteri* strains and that it has the unusual ability to interfere with TNF-alpha mediated propagation of inflammatory responses in human macrophages. Lipocalin-2 and calprotectin are both markers for intestinal inflammation and can be measured in faeces (11, 12). Another lactobacillus strain (rhamnousus GG) has been demonstrated to increase bone mass in mice by increasing serum levels of butyrate, which act on regulatory T-cells, resulting in increased bone anabolic ligand Wnt10b(13). Lactobacillus reuteri has been shown to reduce the suppression of bone derived Wnt10b in type1 diabetic mice(14). Thus, some evidence suggests that lactobacillus reuteri could modify intestinal inflammation and possibly the formation of bone anabolic ligands, such as Wnt10b.

We recently investigated the effect of *L. reuteri* 6475 in a randomized, double-blind, placebo-controlled study, in which 90 older women were included. Treatment was found to reduce bone loss, defined by loss of total volumetric bone mineral density (BMD) in the distal tibia, by half compared to placebo (15). Similar, but in general non-significant, effects were observed at other bone sites (secondary outcomes). However, the effect of *L. reuteri* 6475 has not previously been investigated in early postmenopausal women, and it is not known if the effect of supplementation remains over time, i.e. if the differences between treatment and placebo groups continue to increase over time. Although, it could be anticipated that treatment effects would be similar at the distal tibia and lumbar spine (both sites rich in trabecular bone with high turnover), the effect of *L. reuteri* 6475 supplementation has not been demonstrated on bone loss at the spine, a clinically relevant site to evaluate BMD.

INVESTIGATIONAL PLAN AND PROCEDURES

Study design

The planned study is a double blind, randomized, placebo-controlled, single centre clinical trial. The study, including the pre-specified aims, outcomes, and analysis plan, will be disclosed and registered on *clinicaltrials.gov* prior to study start. By advertising in newspapers, in shops, health clubs, in public places and on social media platforms, and by contacting women (by mail and phone) identified publicly available websites (e.g. www.ratsit.se), we will recruit a minimum of 216 women in the greater Gothenburg and Mölndal area. We aim to include postmenopausal women who have stopped menstruating within the last 1-4 years. The screening procedure will start with the study subject receiving information about the trial, and a short interview to determine eligibility by assessing medical history. If inclusion criteria are fulfilled and no exclusion criteria exist, study subjects will be asked to consent to participate. Informed consent (oral and written) will be required for all participating women. Blood samples will be collected to determine the vitamin D status. Individuals with a 25-hydroxy-vitamin D levels below 25 nmol/L (vitamin D deficiency) will be excluded and individuals with values between 25 and 50 µmol/L (vitamin D insufficiency) will be given a bolus dose of vitamin D (50.000 IU if serum 25-OH vitamin D is 25-35 nmol/L and 30.000 IU if levels are 36-49 nmol/L) prior to

inclusion into the study. Eligible women will undergo bone densitometry of the hip and spine using dual-energy x-ray absorptiometry (DXA) and if BMD and the other requirements are met, inclusion can proceed. Individuals with osteoporosis according to the DXA exam (T-score ≤ -2.5 SD in total hip or spine) together with elevated risk of fracture (defined as >20% risk of osteoporotic fracture within the next ten years according to FRAX), as well as those with serious osteoporosis (T-score < -3 SD in the femoral neck, total hip or spine) will be excluded and referred to their general practitioner for osteoporosis evaluation and potentially medical treatment. After inclusion, women will be randomized (double-blind) to one out of three treatment arms (with at least 72 women in each arm, maximum 80 per arm), where two being different doses of the active treatment and one being placebo. The randomization visit will take place within 4 weeks of the screening visit. Randomization will be carried out by the supplier of *L. reuteri* 6475 (BioGaiaAB) and performed in blocks of 8 study subjects will be generated using the Web site Randomization.com (http://www.randomization.com). The investigators will not have access to the randomization code and blinding will be maintained until study end and database lock. Capsules of freeze-dried *L. reuteri* 6475 (BioGaia AB, Stockholm, Sweden) of either the dose $5x10^8$ or $5x10^9$ colony-forming units (CFU) mixed with maltodextrin powder, taken twice daily, yielding a total daily dose of either $1x10^9$ or $1x10^{10}$ CFU/day respectively, or to placebo which consists of maltodextrin powder only. All capsules will also include 200 IU of cholecalciferol. Once included, all women will fill out a standardized questionnaire regarding life-style habits (e.g., smoking, physical activity, and calcium intake) as well as medical and drug history, food intake, risk factors for osteoporosis and fracture, Food Frequency Questionnaire (FFQ), exercise habits (IPAQ), and gastrointestinal symptoms (GSRS). Included women will be asked to come to quarterly visits to report compliance and possible adverse events, and to bring back remaining and receive new study product. At the 12- and 24-month visits, women will undergo bone densitometry of the hip, spine, and total body with DXA, donate blood, serum, and feces, as well as fill out questionnaires. Continuous registration of possible adverse events will be performed. The bone microstructure, geometry, and volumetric bone mineral density (vBMD) of the tibia will be measured

using high-resolution peripheral quantitative computed tomography (HRpQCT, XtremeCT, Scanco, Switzerland) at baseline and at 12 months and 24 months.

Objectives

Primary outcome

The primary objective is to determine whether dietary supplementation, both high and low dose, with *L. reuteri* 6475 is able to reduce bone loss in tibia total volumetric bone mineral density (relative change) after 2 years of treatment compared to placebo.

Secondary outcomes

Secondary objectives are relative change in after 24 (secondary first) and 12 (secondary last) months in

- Areal bone mineral density (aBMD) at the lumbar spine (DXA)
- Areal BMD of the total hip (DXA)
- Tibia trabecular bone volume fraction (HRpQCT)
- Tibia cortical area (HRpQCT)
- Tibia cortical volumetric BMD (HRpQCT)
- Tibia total volumetric BMD (12 months; HRpQCT)
- Bone formation marker PN1P (blood)
- Bone resorption marker CTX (serum)
- Fecal calprotectin concentration
- Fecal lipocalin-2 concentration
- Serum butyrate concentration
- Serum Wnt10b concentration

SUBJECTS AND METHODS

Study Product and Dosage

L. reuteri 6475, consisting of *L. reuteri* ATCC PTA 6475 will be delivered as capsules in two different doses of $5x10^8$ CFU and $5x10^9$ CFU combined with 200 IU cholecalciferol. One dose is to be taken in the morning and one in the evening yielding a total daily dose of either $1x10^9$ or $1x10^{10}$ CFU/day. The placebo product will also contain vitamin D and be identical to the active product but lack *L. reuteri* ATCC PTA 6475. The study products will be taken twice daily throughout the entire

study-period. The participants will receive sufficient study product for 3 months at baseline, and at the following visits at 3, 6, 9, 12, 15, 18, and 21 months after baseline. The study products shall be kept refrigerated at all times during the study both by the investigators and by the participants. BioGaia AB will provide the study products.

Intervention in normal diet, medications and probiotic supplements

The subjects will be asked to continue with their normal dietary habits during the study period. All participating women will be asked to refrain from consuming any supplements (other than the study product) containing vitamin D or probiotics. Women who use oral glucocorticoids for more than 2 weeks, use osteoporosis medication (bisphosphonates, denosumab or teriparatide), or consume other probiotic supplements will be excluded from the per protocol analysis.

Eligibility

Inclusion criteria

- Postmenopausal women, 45 years or older, within 1-4 years from their last menses.
- Vitamin D levels above 25 nmol/L.
- Signed informed consent.
- Stated availability throughout the entire study period.
- Ability to understand study instructions and willingness to adhere to the protocol.

Exclusion criteria

- Bone mineral density of < -2.5 combined with a FRAX-score of 20% or higher for major osteoporotic fracture.
- Severe osteoporosis, defined as bone mineral density of < -3.0 in either the total hip, femur neck or lumbar spine L1-L4.
- Vertebral fracture (grade II or III) diagnosed using lateral spine imaging with DXA.

- Untreated hyperthyroidism or hyperthyroidism within the last 5 years.
- Known untreated hyperparathyroidism.
- Rheumatoid arthritis.
- Diagnosed with disease causing secondary osteoporosis, including chronic obstructive pulmonary disease, inflammatory bowel disease, celiac disease, or diabetes mellitus.
- Recently diagnosed malignancy (within the last 5 years).
- Oral corticosteroid use.
- Previous (within the last 5 years) use of antiresorptive therapy, including systemic hormone therapy (estrogen), bisphosphonates, strontium ranelate or denosumab.
- Systemic skeletal disease (including e.g. Paget's disease and osteogenesis imperfecta).
- Any systemic disease that could affect bone loss, as judged by the investigator.
- Use of teriparatide (current or during the last 3 years).
- Participation in other clinical trials.
- Current antibiotics treatment or within the last 2 months prior to inclusion.
- Current and within the past 2 months use of probiotic supplement
- Vitamin D deficiency (25-OH vitamin D<25 nmol/l)
- Hypo- or hypercalcemia.
- Osteosynthesis materials in both lower legs (tibia).

Analyses

A standardized questionnaire will be used to collect information at baseline about smoking habits, calcium intake, medical history (e.g. stroke, rheumatoid arthritis and diabetes), medications and previous fractures. Current physical activity and diet habits will be assessed by self-reported questionnaires (IPAQ and FFQ) at baseline and at visits at 12 and 24 months. Daily intake of calcium will be calculated from the questionnaires about calcium-containing foods (e.g. dairy products, vegetables etc). Gastrointestinal symptoms will be analysed using the GSRS questionnaire.

Bone measurements

Areal BMD (aBMD, g/cm^2) will be measured at the hip, lumbar spine (L1-L4) and total body using a GE Lunar iDXA (GE Lunar, Madison, WI, USA). The coefficient of variation (CV) for these measurements range from 0.5 to 3.0% (16). All spine L1-L4 and total hip DXA scans will be analysed by the same certified bone densitometry (ISCD) technologist. Lateral spine imaging will be used to diagnose vertebral compression fractures.

Volumetric BMD (vBMD) and bone microstructure will be investigated at the distal tibia with a high-resolution 3D peripheral quantitative computed tomography (HR-pQCT) device (XtremeCT, Scanco medical AG, Brüttisellen, Switzerland) with a protocol described earlier (17). After processing the images, as described earlier (18), the following parameters will be obtained: total volumetric BMD (vBMD, mg/cm^3), cortical cross-sectional area (CSA, mm^2), cortical volumetric BMD (vBMD, mg/cm^3), trabecular bone volume fraction (BV/TV, %). The CV's range from 0.1% to 1.6% for the tibia measurements (19) in our clinic.

Serum, plasma and blood analyses

Serum and plasma samples will be collected in the morning (fasting), frozen, and stored at -80° C until further analyses. Analyses of 25-OH vitamin D and serum/plasma calcium will be performed directly after blood sample collection. A maximum of 80 ml blood will be collected. Analyses of serum or plasma and bone markers will be performed using commercially available immunosorbent assays. Analyses of the bone anabolic ligand, Wnt10b, will be performed using enzyme-linked immunosorbent assays (ELISA).

Markers of intestinal inflammation

Concentrations of lipocalin-2 and calprotectin, both markers of intestinal inflammation, will be measured in faeces using ELISA.

Analyses of gut microbiota – exploratory analyses

We will isolate DNA from faeces samples using a well-validated protocol and perform PCR to amplify the variable regions (V4) of the bacterial 16*S* rRNA gene

using barcoded primers. Sequencing will be performed on in-house MiSeq equipment. The resulting sequences will be analyzed using QIIME, which is a routine analysis in our laboratory. In addition to assigning sequences to "species-level" operational taxonomic units (OTUs) using a 97% pairwise-identity we will estimate microbial diversity within communities. To this end we will employ rarefaction plots and phylogenetic diversity measures (weighted and unweighted UniFrac distance metrics) to compare diversity between samples. This approach will reveal which bacterial species are present in the gut of patients before and after treatment and if women responding to treatment have a different metagenome than women not responding. Metagenome analyses of all samples will also be performed. For shotgun metagenomics sequencing DNA fragments of approximately 300 bp will be sequenced on an Illumina NextSeq 500 instrument (150 bp; paired-end) at Genomics Core Facility at the Sahlgrenska Academy, University of Gothenburg. Genes will be annotated to KEGG orthologies (KOs) and taxonomic and KO composition be analyzed using an updated version of the MEDUSA pipeline(20), in which the raw reads will be trimmed by FASTX (http://hannonlab.cshl.edu/fastx_toolkit/; with a quality threshold of 20 and minimum length of 35 bp), filtered to remove human reads (version hg19), and then mapped to bacterial gene and genome catalogues using Bowtie2 (21). An additional filter will be applied during the mapping process containing reads with at least 95% identity to obtain high-quality reads. The obtained taxonomic composition and KO profile matrix will be further analyzed byDESeq2 package (version 1.4.5) (22). Pathway enrichment analyses will be based on KEGG annotation (23) and hypergeometric test using goseq (24). We have significant experience in DNA extractions (>10,000 fecal samples have been extracted) and metagenomics analyses (25).

Analyses of metabolites – exploratory analyses

Plasma, taken after overnight fast, at each and every yearly visit, will also undergo analysis of up to 1000 known metabolites including butyrate (Metabolon Inc., NC, USA). Metabolon Inc. will identify metabolites and fatty acids using their DiscoveryHD4 platform in addition to their Fatty Acid Metabolism (FAME) panel, utilizing a combination of ultra-high-performance liquid chromatography with tandem

mass spectrometry and gas chromatography. Only samples from women who adhere to study protocol will be analysed. Pathway analysis will be employed to identify specific signalling systems (e.g. butyrate emanating from intestinal bacteria) affected by treatment. Using 100 µl serum or plasma, this technique is able to measure metabolite quantities with approximately 5% coefficient of variation. Ratios of metabolite concentrations will be created for each study subject by dividing each respective metabolite concentration analyzed at year 1, 2 and 3 with the baseline concentration. Metabolites which are affected by L. reuteri supplementation will also be investigated in relation to changes (over 3 years) in spine BMD, which would indicate but not determine a causal relationship.

Adherence

Subjects will be asked to return the used packages from the study products to allow assessment by the investigator of adherence. The investigators will also assess adherence by contacting study subjects every week for the first 4 weeks, followed by once monthly contacts by phone. Adherence to protocol will be defined as an intake of >80% of prescribed doses.

Ethical review and informed consent

This protocol will be submitted by the PI to the appropriate ethical committee for review and approval must be obtained prior to commencement of the study.
The Declaration of Helsinki will be followed. A written informed consent from all subjects must be obtained prior to inclusion of any study subject.

Study period

It is anticipated that the study will start during the third quarter of 2019 and recruitment should be completed within 6-8 months after study start.

Study time line

Activity	**Day 0**	**Rando mis.**	**3 months**	**6 months**	**9 months**	**12 months**	**15 months**	**18 months**	**21 months**	**24 months**
Serum 25-OH vitamin D, calcium	X									
DXA, (total hip, total body and lumbar spine L1-L4)	X					X				X
S-CTX, S-P1NP	X					X				X
HR-pQCT tibia	X					X				X
Serum, plasma, faeces samples	X					X				X
Adverse events (continuous monitoring)			X	X	X	X	X	X	X	X
Questionnaires (medical history etc)	X					X				X
Distribution of study product		X	X	X	X	X	X	X	X	

Monitoring

During the study, the clinical research manager from BioGaia AB will have regular contact with the PI investigational site. Study personal will contact study subjects every week during the first 4 weeks, followed by once monthly, to monitor compliance and register any side effects of treatment.

Case Report Forms

CRFs will be used throughout the study to record potential adverse events.

Changes to the Study Protocol

Amendments are to be written and signed by Principal Investigator.

Data analysis

Data is to be compiled and analysed by the Principal Investigator.

Statistical analyses

A detailed statistical analysis plan (SAP) will be developed by Statistiska konsultgruppen, describing all planned analysis prior to unblinding. The primary endpoint for analyses is 24-month relative change in tibia volumetric BMD measured with HRpQCT. The expected effects of supplementation are described in Table 2

Variable	L.reuteri 6475 high dose (G1)	L.reuteri 6475 low dose (G2)	Placebo (G3)	Drop-out
Tibia total vBMD (ttvBMD) at baseline	230 ± 40	230 ± 40	230 ± 40	
% change in ttvBMD at one year (Secondary)	-0.90 ± 1.6	-1.08 ± 1.6	-1.85 ± 1.6	10%
% change in ttvBMD BMD at two years (Primary)	-1.75 ± 1.6	-2.10 ± 1.6	-3.50 ± 1.6	15%
SD = 1.6 (variance 2.56) is divided into 1.52 for between individuals' SD (variance 2.30) and 0.51 for within individual SD (variance 0.26). The total variance (0.26+2.30) agrees with the assumed 3.56 and ICC of ~0.90 (2.30/2.56) agrees with the observed in the first LR6475 study (ELBOW) performed on older women. In a similar way, SD of 2 is divided into 1.90 for between-subject SD and 0.63 for within subject SD.				

A smaller group to group difference is anticipated using the low-dose (G2) supplementation. For the power calculation, data was simulated according to the above table for 1000 studies. For each study, 10% and 15% missing data was assumed at 1 and 2 years, respectively. The analysis is performed with a mixed model for repeated measurements with time, group, time x group and baseline value as fixed effects and multiple imputation based on sampling of 50. Statistical power is based on 0.05 or 0.025 with Bonferroni-correction. The primary outcome (confirmed analysis) will be relative change in tibia total vBMD at 24 months. Using Bonferroni correction, the statistical power will be:

- G1 vs G3 results in power ≥99.9%
- G2 vs G3 results in power ≥99.9%

Secondary outcomes will be spine BMD, total hip BMD, tibia total volumetric BMD, cortical area, trabecular bone volume fraction, bone turnover markers (serum CTX and P1NP) at 24 months and at 12 months. At 12 months, total vBMD will also be a secondary analysis. For relative change in tibia total vBMD at 12 months the power is estimated to:

- G1 vs G3 results in power 95.0%
- G2 vs G3 results in power 81.9%

Assuming an alpha of 0.05.

For relative change in spine BMD at 24 months the power is estimated to:

- G1 vs G3 results in power 99.8%
- G2 vs G3 results in power 86.8%

Primary analysis will be intention to treat (ITT) but also per protocol (PP) analysis will be performed and reported.

Final Study Report

A final report is to be prepared as soon as possible after the last subject has completed the trial.

Indemnity

The principal investigator carries individual medical liability insurance. The hospital also indemnifies the investigators in approved research. Injury adjudicated to be caused by the study product under trial is the responsibility of the producer (BioGaia AB). BioGaia AB carries insurance for clinical trials with a limit of 1 MSEK per individual and 10 MSEK in total.

SAFETY CONSIDERATIONS

Scientific background

Lactobacillus reuteri (*L. reuteri*) was originally described as *Lactobacillus fermentum* type II (26), but is now recognised as a distinctive species (27). *L. reuteri* is a heterofermentative species that resides in the gastrointestinal (GI) tract of humans and animals (28) and is considered to be one of the few true autochthonous (indigenous) *Lactobacillus* species in man (6). The probiotic mode of action of *Lactobacilli* and *L. reuteri* in particular is generally attributed to the ability to exert inhibitory effect of pathogenic microorganisms by a combination of different mechanisms including excretion of lactic acid, hydrogen peroxide, antimicrobial substances and bacteriocins (28). *L. reuteri*, like other lactic acid bacteria, is able to convert milk sugar (lactose) into lactic acid and has been shown to produce hydrogen peroxide. In addition to lactic acid, *L. reuteri* ferments carbohydrates into short chain fatty acids (e.g., acetic acid), which has an antibacterial effect.

Axelsson et al. (29) reported that *L. reuteri* converted glycerol into a potent, broad-spectrum antimicrobial that was termed "reuterin" that was capable of inhibiting growth of species representing several bacterial genera including *Escherichia*, *Salmonella*, *Shigella*, *Proteus*, *Pseudomonas*, *Clostridium* and *Staphylococcus* as well as yeasts, fungi, protozoa, and viruses. Reuterin is excreted by *L. reuteri* during anaerobic growth in the presence of glycerol (30). Chung et al. (31) showed that reuterin was synthesised under environmental conditions similar to those which exist in the regions of the GI tract where *L. reuteri* has been isolated, although *in vivo* production of reuterin has not been demonstrated. Reuterin synthesis was stimulated

by contact with other bacteria, that to a varying degree are found in the human gut, such as *E. coli*, *Salmonella typhimurium*, *Shigella*, *Proteus*, *Pseudomonas fluorescens*, *Staphylococcus epidermidis*, *Bacillus megaterium*, *Clostridium sporogenes*, *Pediococcus pentosaceus*, *Leuconostoc mesenteroides*, and *Streptococcus cremoris*. Although *L. reuteri* exerts a strong antibiotic action on GI tract pathogens, it does not negatively affect the normal, beneficial microflora (28, 32, 33)).

Lactobacillus reuteri is widely used as a probiotic dietary supplement. Milk supplemented with *L. reuteri* was first introduced in Sweden in 1991. Currently, dairy products containing *L. reuteri* are sold in the US, Finland, Japan, Korea, Spain, Portugal and the UK. In 2000, chewable tablets containing *L. reuteri* were introduced in the US and these tablets have since been introduced in Europe, S. Africa and Asia. To date the equivalent of more than 1 billion doses of 10^8 CFU *L. reuteri* have been sold through the various products in some 18 countries worldwide. No clinical infection or untoward side-effect involving *L. reuteri* has ever been reported.

Other *Lactobaillus* species have been linked to rare case reports of infection, in which the patients were either immunocompromised or otherwise suffering from other severe clinical conditions (34, 35). There are no reports of isolation of *L. reuteri* in connection with any disease or pathological process in either animals or humans. Indeed, an excellent study recently reported on bacteremia in the Finnish population (36) did not detect any cases of *L. reuteri* in the circulation, although several other *Lactobacillus* species were detected in the blood. These observations were made during a period when *L. reuteri* was widely consumed in Finland in dairy and juice products.

Wolf et al. studied the safety and tolerance of *L. reuteri* ingestion in healthy males in a randomized, double-blinded placebo controlled trial (37). Thirty healthy males (n=15/group) were randomly assigned to receive either *L. reuteri* ($1x10^{11}$) or placebo capsules for 21 days. The incidence of subjective tolerance factors such as flatulence, diarrhoea and cramping were infrequent and similar between the groups. The results indicate that *L. reuteri* can be ingested at a level of $1x10^{11}$ CFU/day without any

clinically significant safety or tolerance problems. Further, Wolf et al. (1998) examined the safety and tolerance to *L. reuteri* in individuals with HIV infection in a randomized, double-blinded placebo controlled trial (38). The subjects were supplemented either with *L. reuteri* (10^{10} CFU/day, n=15) or placebo (n=20) capsules for 21 days. These authors concluded that immunocompromised individuals can ingest *L. reuteri* at $1x10^{10}$ CFU/day without any clinical safety and tolerance problems.

Ruiz-Palacios et al. established the tolerance and dose response of a probiotic mixture containing *L. reuteri*, *L. acidophilus* and *B. infantis* in children (n=72), ages 12 to 36 months (39). The children were randomly assigned to one of three different amounts of daily supplementation with *L. reuteri* together with unchanged levels *L. acidophilus* and *B. infantis* or non-probiotic placebo. No significant differences in the incidence of vomiting, abdominal discomfort, gas, and stool characteristics were observed among the groups. *L. reuteri* supplementation was well tolerated by the children up to a dose of $1x10^{10}$ CFU/day. In further studies in 248 children (aged 12 to 35 months) children receiving a probiotic blend including *L. reuteri* for 14-16 weeks, no adverse health effects were reported (39).

Shornikova et al. studied children (ages 6 to 36 months) with infectious diarrhoea who were treated with *Lactobacillus reuteri*. In one of the studies, children received 10^{10} to 10^{11} CFU of *L. reuteri* once per day, and in the other 10^7 or 10^{10} CFU/day for 5 days. No adverse effects of *L. reuteri* supplementation on weight gain, consumption of oral rehydration solution or electrolyte, or on acid-base balance could be detected (40, 41).

Karvonen et al. (2001) studied the safety of doses of *L. reuteri* between 10^5 to 10^9 CFU/day given to 90 healthy neonates (as powder additive to breast milk) for 28 days (42). No difference in symptoms like abdominal discomfort, abdominal pains or cramps were observed compared to placebo. Healthy infants between 3-65 days of age were given *L. reuteri* -supplemented infant formula (approx 10^8 CFU/day) or a placebo infant formula for 4 weeks (43). The groups were similar with regard to growth, gastrointestinal function and well-being of the infants. Supplementation with

L. reuteri in infants from birth up to 12 months was found to be well-tolerated and there were no elevations in blood D(-)-lactic acid levels during supplementation (44). Thus, *L. reuteri* is considered a safe food supplement to give to humans of all ages.

Expected safety profile of *L. reuteri* ATCC PTA 6475

The *L. reuteri* ATCC PTA 6475 strain is a naturally occurring strain of human origin. It is a typical member of the *L. reuteri* species, with typical sugar fermentation patterns, reuterin production and growth characteristics. Identification using 16SrRNA gene analysis shows that it has 99% similarity with the type strain of *L. reuteri* thus confirming that it belongs to this well-known and well-studied species that is considered safe for human consumption. In a prior study performed by Nilsson, AG et al., the specific strain, *L. reuteri* ATCC PTA 6475 was well tolerated by elderly women and reduced bone loss by half in osteopenic women compared to women treated with placebo. The proportion of adverse events was similar between groups and no serious adverse events related to the product were reported (15). In vitro studies indicate that this strain has strong tolerance to acid environments, as do many other *L. reuteri* strains. Both strains used in this study are deposited in public culture collections.

Antibiotic resistance profiles have been made on *L. reuteri* ATCC PTA 6475 and it displays typical patterns of resistance that are intrinsic for the *L. reuteri* species (45). As essentially all hetero-fermenting lactobacilli, *L. reuteri* strains have a natural, non-transferable resistant to vancomycin. These strains are sensitive to many antibiotics including, streptomycin and erythromycin.

To date, despite extensive animal and human investigation of the effects of supplementation of the diet with live *L. reuteri* strains of various origins since the 1980s, no possible serious adverse events have been reported. No serious adverse events were reported for the *L. reuteri* ATCC PTA 6475 in elderly women and it is therefore no reason to expect an unusual safety profile of *L. reuteri* ATCC PTA 6475 in younger postmenopausal women.

Other safety considerations

The principal investigator is responsible for monitoring the safety of subjects who have entered the study. All adverse events (AE) occurring after any administration of the study product will be followed to the end of the study or until resolution. AE will be evaluated by the Investigator and reported in the CRF.
Serious adverse events, as defined below, must be reported to the producer within 24 hours of when the investigator became aware of the SAE. It is also important to report all adverse events that result in permanent discontinuation of the study product, whether serious or non-serious.

Adverse Events

An adverse event (AE) is any undesirable medical occurrence in a subject administered a study product, regardless of causality assessment. An adverse event can therefore be any unfavourable and unintended sign (including an abnormal laboratory finding), symptom, or disease temporally associated with the use of a study product, whether or not considered related to the study product. All adverse events, including observed or volunteered problems, complaints, or symptoms are to be recorded on the case report form (CRF). Each adverse event is to be evaluated for duration, intensity, and causal relationship with the study product or other factors. Subjects should be instructed to report any AE that they experience to the Investigator. Investigators should assess for AE at each visit. AE occurring during the clinical trial, including the follow-up period should be recorded on the appropriate AE CRF. To capture the most potentially relevant safety information during a clinical trial, it is important that investigators record accurate AE terms on CRF.
If discernible at the time of completing an AE in CRF, a specific disease or syndrome rather than individual associated signs and symptoms should be identified by the investigator and recorded on the CRF. However, if an observed or reported sign or symptom is not considered a component of a specific disease or syndrome by the investigator, it should be recorded as a separate AE on the CRF.

Serious Adverse Events

A serious adverse event (SAE) is any AE that

- results in death.
- is immediately life threatening.
- requires or prolongs hospitalisation.
- results in persistent or significant disability/incapacity.
- is a medically significant event for any reason.

Reporting Serious Adverse Events

All SAE must be reported to the producer of *L. reuteri* study products within one day from when the investigator became aware of the SAE. This can be done by faxing a completed SAE Report Form or by direct telephone communication.
A completed SAE Report Form should follow all telephone reports. The BioGaia personnel are available for SAE reporting Monday through Friday at office hours.
Contact information:

BioGaia AB
P.O. Box 966
SE-220 09 Lund
SWEDEN
Phone: +46 46 311 915
Mobile: +46 709 380 350
Fax: + 46 46 311 901
e-mail: az@biogaia.se

References

1. Johnell O, Kanis J. Epidemiology of osteoporotic fractures. Osteoporos Int. 2005;16 Suppl 2:S3-7.
2. Vis M, Guler-Yuksel M, Lems WF. Can bone loss in rheumatoid arthritis be prevented? Osteoporos Int. 2013;24(10):2541-53.
3. Rizzoli R. Postmenopausal osteoporosis: Assessment and management. Best Pract Res Clin Endocrinol Metab. 2018;32(5):739-57.

4. Adams MR, Marteau P. On the safety of lactic acid bacteria from food. International journal of food microbiology. 1995;27(2-3):263-4.
5. Reid G, Kim SO, Kohler GA. Selecting, testing and understanding probiotic microorganisms. FEMS immunology and medical microbiology. 2006;46(2):149-57.
6. Reuter G. The Lactobacillus and Bifidobacterium microflora of the human intestine: composition and succession. Current issues in intestinal microbiology. 2001;2(2):43-53.
7. Connolly E. Lactobacillus reuteri ATCC 55730; a clinically proven probiotic. Nutrafoods. 2004;3:15-22.
8. Valeur N, Engel P, Carbajal N, Connolly E, Ladefoged K. Colonization and immunomodulation by Lactobacillus reuteri ATCC 55730 in the human gastrointestinal tract. Applied and environmental microbiology. 2004;70(2):1176-81.
9. McCabe LR, Irwin R, Schaefer L, Britton RA. Probiotic use decreases intestinal inflammation and increases bone density in healthy male but not female mice. Journal of cellular physiology. 2013;228(8):1793-8.
10. Britton RA, Irwin R, Quach D, Schaefer L, Zhang J, Lee T, et al. Probiotic L. reuteri treatment prevents bone loss in a menopausal ovariectomized mouse model. Journal of cellular physiology. 2014;229(11):1822-30.
11. Derikx JP, Luyer MD, Heineman E, Buurman WA. Non-invasive markers of gut wall integrity in health and disease. World J Gastroenterol. 2010;16(42):5272-9.
12. Moschen AR, Adolph TE, Gerner RR, Wieser V, Tilg H. Lipocalin-2: A Master Mediator of Intestinal and Metabolic Inflammation. Trends Endocrinol Metab. 2017;28(5):388-97.
13. Tyagi AM, Yu M, Darby TM, Vaccaro C, Li JY, Owens JA, et al. The Microbial Metabolite Butyrate Stimulates Bone Formation via T Regulatory Cell-Mediated Regulation of WNT10B Expression. Immunity. 2018;49(6):1116-31 e7.
14. Zhang J, Motyl KJ, Irwin R, MacDougald OA, Britton RA, McCabe LR. Loss of Bone and Wnt10b Expression in Male Type 1 Diabetic Mice Is Blocked by the Probiotic Lactobacillus reuteri. Endocrinology. 2015;156(9):3169-82.
15. Nilsson AG, Sundh D, Backhed F, Lorentzon M. Lactobacillus reuteri reduces bone loss in older women with low bone mineral density: a randomized, placebo-controlled, double-blind, clinical trial. J Intern Med. 2018.
16. Johansson H, Oden A, Lerner UH, Jutberger H, Lorentzon M, Barrett-Connor E, et al. High serum adiponectin predicts incident fractures in elderly men: Osteoporotic fractures in men (MrOS) Sweden. J Bone Miner Res. 2012;27(6):1390-6.
17. MacNeil JA, Boyd SK. Improved reproducibility of high-resolution peripheral quantitative computed tomography for measurement of bone quality. Med Eng Phys. 2008;30(6):792-9.
18. Laib A, Hauselmann HJ, Ruegsegger P. In vivo high resolution 3D-QCT of the human forearm. Technol Health Care. 1998;6(5-6):329-37.
19. Rudang R, Darelid A, Nilsson M, Mellstrom D, Ohlsson C, Lorentzon M. X-ray-verified fractures are associated with finite element analysis-derived bone

strength and trabecular microstructure in young adult men. J Bone Miner Res. 2013;28(11):2305-16.
20. Karlsson FH, Nookaew I, Nielsen J. Metagenomic data utilization and analysis (MEDUSA) and construction of a global gut microbial gene catalogue. PLoS Comput Biol. 2014;10(7):e1003706.
21. Langmead B, Salzberg SL. Fast gapped-read alignment with Bowtie 2. Nat Methods. 2012;9(4):357-9.
22. Love MI, Huber W, Anders S. Moderated estimation of fold change and dispersion for RNA-seq data with DESeq2. Genome Biol. 2014;15(12):550.
23. Kanehisa M, Goto S. KEGG: kyoto encyclopedia of genes and genomes. Nucleic Acids Res. 2000;28(1):27-30.
24. Young MD, Wakefield MJ, Smyth GK, Oshlack A. Gene ontology analysis for RNA-seq: accounting for selection bias. Genome Biol. 2010;11(2):R14.
25. Karlsson FH, Fak F, Nookaew I, Tremaroli V, Fagerberg B, Petranovic D, et al. Symptomatic atherosclerosis is associated with an altered gut metagenome. Nat Commun. 2012;3:1245.
26. Reuter G. Das vorkommen von laktobacillen in lebensmitteln und ihr verhalten im menschlichen intestinaltrakt. Zbl Bak Parasit Infec Hyg I Orig. 1965(197 S):468-87.
27. Kandler O, Stetter K, Kohl R. Lactobacillus reuteri sp. nov. a new species of heterofermentative lactobacilli. Zbl Bakt Hyg Abt Orig. 1980(C1):264-9.
28. Casas I, Dobrogosz WJ. Validation of the probiotic concept: Lactobacillus reuteri confers broad spectrum protection against disease in humans and animals. . Microbial Ecology in Health and Disease. 2000;12:247-85.
29. Axelsson L, Chung T, Dobrogosz WJ, Lindgren S. Production of a broad spectrum antimicrobial substance by Lactobacillus reuteri. Microbial Ecology Health Disease 1989;2:131-6.
30. Talarico TL, Casas IA, Chung TC, Dobrogosz WJ. Production and isolation of reuterin, a growth inhibitor produced by Lactobacillus reuteri. Antimicrobial agents and chemotherapy. 1988;32(12):1854-8.
31. Chung T, Axelsson L, Lindgren S, Dobrogosz WJ. In vitro studies on reuterin synthesis by Lactobacillus reuteri. . MicrobEcolHealth Dis. 1989;2:137-44.
32. Lorca GL, Wadstrom T, Valdez GF, Ljungh A. Lactobacillus acidophilus autolysins inhibit Helicobacter pylori in vitro. Current microbiology. 2001;42(1):39-44.
33. Jacobsen CN, Rosenfeldt Nielsen V, Hayford AE, Moller PL, Michaelsen KF, Paerregaard A, et al. Screening of probiotic activities of forty-seven strains of Lactobacillus spp. by in vitro techniques and evaluation of the colonization ability of five selected strains in humans. Applied and environmental microbiology. 1999;65(11):4949-56.
34. Andriessen MP, Mulder JG, Sleijfer DT. Lactobacillus septicemia, an unusual complication during the treatment of metastatic choriocarcinoma. Gynecologic oncology. 1991;40(1):87-9.
35. Aguirre M, Collins MD. Lactic acid bacteria and human clinical infection. The Journal of applied bacteriology. 1993;75(2):95-107.
36. Salminen MK, Tynkkynen S, Rautelin H, Saxelin M, Vaara M, Ruutu P, et al. Lactobacillus bacteremia during a rapid increase in probiotic use of Lactobacillus rhamnosus GG in Finland. Clinical infectious diseases : an official

publication of the Infectious Diseases Society of America. 2002;35(10):1155-60.

37. Wolf B, Garleb K, Ataya D, Casas I. Safety and tolerance of Lactobacillus reuteri in healthy adults male subjects. Microbial Ecology Health Disease. 1995;8:41-50.
38. Wolf BW, Wheeler KB, Ataya DG, Garleb KA. Safety and tolerance of Lactobacillus reuteri supplementation to a population infected with the human immunodeficiency virus. Food and chemical toxicology : an international journal published for the British Industrial Biological Research Association. 1998;36(12):1085-94.
39. Ruiz-Palacios G, Guerrero M, Hilty M, Dohnalek M, Newton J, Calva J, et al. Feeding of a probiotic for the prevention of community-acquired diarrhea in young Mexican children. PediatrRes1996. p. Abstract no. 1089.
40. Shornikova AV, Casas IA, Isolauri E, Mykkanen H, Vesikari T. Lactobacillus reuteri as a therapeutic agent in acute diarrhea in young children. Journal of pediatric gastroenterology and nutrition. 1997;24(4):399-404.
41. Shornikova AV, Casas IA, Mykkanen H, Salo E, Vesikari T. Bacteriotherapy with Lactobacillus reuteri in rotavirus gastroenteritis. The Pediatric infectious disease journal. 1997;16(12):1103-7.
42. Karvonen A, Casas I, Vesikari T. Safety and possible anti-diarrhoeal effect of the probiotic Lactobacillus reuteri after oral administration to neonate. Clin Nutr. 2001;suppl 3:abstract no. 216.
43. Weizman Z, Alsheikh A. Safety and tolerance of a probiotic formula in early infancy comparing two probiotic agents: a pilot study. Journal of the American College of Nutrition. 2006;25(5):415-9.
44. Connolly E, Abrahamsson.T., Bjorksten B. Safety of D(-)-lactic acid producing bacteria in the human infant. Journal of pediatric gastroenterology and nutrition. 2005;41(4):489-92.
45. Egervarn M, Danielsen M, Roos S, Lindmark H, Lindgren S. - Antibiotic susceptibility profiles of Lactobacillus reuteri and Lactobacillus fermentum. J Food Prot. 2007;70(2):412-8.

Mölndal 2022-12-01

Protocol amendment to the ELBOW II Study - Prevention of Early Postmenopausal Bone Loss in Lactobacillus reuteri – A Randomized, Placebo-Controlled, Single Centre Clinical Trial

I. Definition of the per protocol population.

The new definition of the per protocol population: Women who during the study use oral glucocorticoids for more than 2 weeks, use osteoporosis medication (bisphosphonates, denosumab or teriparatide), menopausal hormone therapy, or consume other probiotic supplements will be excluded from the per protocol analysis.

II. Additional secondary outcomes to be analyzed

Secondary objectives are relative change in after 24 (secondary first) and 12 (secondary last) months in

- Plasma acetate
- Plasma propionate

Change in biochemical analyses as per

- Butyrate will be analyzed in plasma, not in serum
- CTX will be analyzed in plasma, not in serum

Serum Wnt10b will be analyzed at a later stage, when appropriate methods are available.

Fecal calprotectin and lipocalin-2 will be analyzed at a later stage and will not be part of the primary analyses.

Mattias Lorentzon,
Principal investigator

Geriatric Medicine
Sahlgrenska University Hospital
Göteborgsvägen 31, 43180 Mölndal
Sweden
