# Supplementary material for: Limosilactobacillus reuteri 6475 and Prevention of Early Postmenopausal Bone Loss: A Randomized Clinical Trial
Source: JAMA Netw Open. 2024 Jun 12;7(6):e2415455. doi: 10.1001/jamanetworkopen.2024.15455 (PMC11170297; doi:10.1001/jamanetworkopen.2024.15455)
Supplement: Supplement 2. — Statistical Analysis Plan [file jamanetwopen-e2415455-s002.pdf]

Statistical Analysis Plan

ELBOW II Study
Prevention of Early Postmenopausal Bone Loss in *Lactobacillus reuteri* – A Randomized, Placebo-Controlled, Single Centre Clinical Trial

Mattias Lorentzon, Principal Investigator

Aldina Pivodic, Statistician

December 7, 2022

Authored by:
Aldina Pivodic
aldina.pivodic@apnc.se
+46 709 47 14 12

Revisions:

Nr	Description	Date

Table of Contents

Abbreviations 4
1 Study Objectives 5

1.1 Primary Objective 5
1.2 Secondary Objectives 5
2 Study Design 5
2.1 Treatment Groups 6
2.2 Flow Chart 6
3 Study Populations 6
3.1 Intention-to-Treat Population 6
3.2 Per Protocol Population 7
3.3 Safety Population 7
4 Study Variables 7
4.1 Baseline Variables 7
4.2 Medical History 8
4.3 Prior Medications 9
4.4 Efficacy Variables 9
4.4.1 Primary Variable 9
4.4.2 Secondary Variables 9
4.5 Safety Variables 10
4.5.1 Exposure and Compliance 10
4.5.2 Adverse Events 10
4.5.3 Quality of Life Variables 10
5 Statistical Methods 11
5.1 Sample Size 11
5.2 General Methodology 12
5.3 Adjustment of Type I Error 13
5.4 Patient Disposition and Datasets Analyzed 13
5.5 Protocol Deviations 13
5.6 Demographics and Baseline Characteristics 14
5.7 Medical History 14
5.8 Prior Medications 14
5.9 Efficacy Analyses 14
5.9.1 Primary Analysis 14
5.9.2 Secondary Analyses 14

5.10 Subgroup Analyses 15
5.11 Safety Analyses 15
5.11.1 Exposure and Compliance 15
5.11.2 Adverse Events 15
5.11.3 Quality of Life 15
6 Interim Analyses 16
7 Changes from Study Protocol 16
8 Planned Tables and Figures 17

Abbreviations

**aBMD**	**Areal bone mineral density**
**AE**	Adverse events
**AIC**	Akaike's information criterion
**CI**	Confidence interval
**FFQ**	Food frequency questionnaire
**GSRS**	Gastrointestinal symptom rating scale
**IPAQ**	International physical activity questionnaire
**ITT**	Intention-to-treat
***L. reuteri* 6475**	*Lactobacillus reuteri* ATCC PTA 6475
**LSM**	Least Square Mean
**PP**	Per protocol
**QoL**	Quality of life
**SD**	Standard deviation
**vBMD**	Volumetric bone mineral density

1 Study Objectives

1.1 Primary Objective

The primary objective of this study is to determine whether dietary supplementation, both high ($1x10^{10}$ CFU/day) and low ($1x10^{9}$ CFU/day) dose, with *Lactobacillus reuteri* ATCC PTA 6475 (*L. reuteri* 6475) is able to reduce bone loss in tibia total volumetric bone mineral density (vBMD) (relative change) after 2 years of treatment compared to placebo.

1.2 Secondary Objectives

Secondary objectives aimed to be confirmed with this study are the following variables:

- Relative change after 1 year and 2 years in areal bone mineral density (aBMD) at the lumbar spine (DXA), both high and low dose vs placebo
- Relative change after 1 year and 2 years in aBMD of the total hip (DXA), both high and low dose vs placebo
- Relative change after 1 year in tibia total vBMD, both high and low dose vs placebo, and trend over time (interaction time x treatment)

Other secondary objectives aimed to be studied exploratively are the following variables, studied after 2 years (first) and 1 year (second):

- Tibia trabecular bone volume fraction (HRpQCT)
- Tibia cortical area (HRpQCT)
- Tibia cortical vBMD (HRpQCT)
- Serum bone formation marker P1NP
- Plasma resorption marker CTX
- Plasma butyrate, acetate, and propionate concentrations
- Fecal calprotectin concentration *(analyzed at a later stage)*
- Fecal lipocalin-2 concentration *(analyzed at a later stage)*
- Serum Wnt10b concentration *(analyzed at a later stage)*

2 Study Design

This study is a double-blind, randomized, placebo-controlled, single center clinical trial comparing both high and low dose of *L. reuteri* 6475 vs placebo in early postmenopausal women followed up for two years. At least seventy-two (72) and a maximum of eighty (80) women were planned to be recruited in each of the three randomized groups.

2.1 Treatment Groups

There are three randomized treatment groups in this study:

1. *L. reuteri* 6475 high dose ($1x10^{10}$ CFU/day)
2. *L. reuteri* 6475 low dose ($1x10^{9}$ CFU/day)
3. Placebo

2.2 Flow Chart

Activity	**Day 0**	**Rand**	**3m**	**6m**	**9m**	**12m**	**15m**	**18m**	**21m**	**24m**
Serum 25-OH vitamin D, calcium	X									
DXA, (total hip, total body and lumbar spine L1-L4)	X					X				X
S-CTX, S-P1NP	X					X				X
HR-pQCT tibia	X					X				X
Serum, plasma, faeces samples	X					X				X
Adverse events (continuous monitoring)			X	X	X	X	X	X	X	X
Questionnaires (medical history etc)	X					X				X
Distribution of study product		X	X	X	X	X	X	X	X	

3 Study Populations

3.1 Intention-to-Treat Population

The intention-to-treat (ITT) population will include all women that were randomized into the study. Analysis will be performed on as-randomized basis.

3.2 Per Protocol Population

The per-protocol (PP) population will include all women that were randomized into the study and that do not have any major protocol violations in the study. The final PP population will be defined in blinded manner, after the clean file of the study database and before the database lock and unblinding of the study treatment.

3.3 Safety Population

The safety population will include all women that were randomized into the study and that have taken at least one dose of the study treatment or placebo.

4 Study Variables

4.1 Baseline Variables

Following baseline variables will be described:

- Age (years)
- Height (cm)
- Weight (kg)
- Body mass index (BMI) (kg/m^2)
- Total body fat (%)
- Appendicular lean mass (kg/m^2)
- Current smoking (n, %)
- Alcohol (≥3 standard drinks per day)
- Parental hip fracture (n, %)
- Run-in period (days)
- Previous glucocorticoids (5mg of Prednisolone or more for 3 months or more) (n, %)
- Tibia ultradistal (standard site) Cortical area (mm)
- Tibia ultradistal (standard site) total volumetric BMD (mg/cm^3)
- Tibia ultradistal (standard site) cortical volumetric BMD (mg/cm^3)
- Tibia ultradistal (standard site) trabecular bone volume fraction (%)
- Bone mineral density (DXA) total hip (g/cm^2)
- Bone mineral density (DXA) femoral neck (g/cm^2)
- Bone mineral density (DXA) lumbar spine (g/cm^2)
- T-score total hip (SD)
- T-score lumbar spine (SD)
- 10-year fracture probability (FRAX) for major osteoporotic fracture (with BMD)
- 10-year fracture probability (FRAX) for major osteoporotic fracture (without BMD)
- 10-year fracture probability (FRAX) for hip fracture (with BMD)

- 10-year fracture probability (FRAX) for hip fracture (with BMD)
- Serum 25-OH-vitamin D (nmol/l)
- Plasma total calcium (mmol/l)
- Gastrointestinal Symptom Rating Scale (GSRS), total score (0-45)
- Energy intake (kcal/day)
- Protein intake (g/day)
- Fat intake (g/day)
- Carbohydrates intake (g/day)
- Fiber intake (g/day)
- Salt intake (g/day)
- Calcium intake (mg/day)
- Physical activity (IPAQ; METs per week)

4.2 Medical History

Following medical history will be described:

- Diabetes (n, %)
- Hyperthyroidism (n, %)
- Hypothyroidism (n, %)
- Osteoporosis (n, %)
- Chronic bronchitis, asthma or emphysema (n, %)
- Stroke (n, %)
- Hypertension (n, %)
- Myocardial infarction (n, %)
- Angina (n, %)
- Heart failure (n, %)
- Chronic liver disease (n, %)
- Osteoarthritis (n, %)
- Gout (n, %)
- Muscle rheumatism (n, %)
- Cancer (n, %)
- Prevalent falls (during the last year, n (%))
- Prevalent fracture (n, %)
- Age at menarche (years)
- Age at menopause (years)
- Years since menopause (yrs)

4.3 Prior Medications

Medications for the treatment of

- Hypothyroidism (n, %)
- Osteoporosis (n, %)
- Hypertension (n, %)
- Angina (n, %)
- Heart failure (n, %)
- Chronic bronchitis, asthma or emphysema (n, %)
- Liver disease (n, %)
- Oral glucocorticoids (n, %)
- Osteoarthritis (n, %)
- Calcium and vitamin D supplements

4.4 Efficacy Variables

4.4.1 Primary Variable

This study's primary variable is:

- Relative change in tibia total vBMD at 2 years, high dose vs placebo and low dose vs placebo

4.4.2 Secondary Variables

This study's secondary variables aimed to be confirmed are the following:

- Relative change in spine aBMD at 2 years and 1 year, high dose vs placebo and low dose vs placebo
- Relative change in total hip aBMD at 2 years and 1 year, high dose vs placebo and low dose vs placebo
- Relative change in tibia total vBMD at 1 year, high dose vs placebo, low dose vs placebo and trend over time (interaction time x treatment)

This study's secondary variables aimed to be studied exploratively are the following:

- Relative change in tibia trabecular bone volume fraction at 2 years and 1 year, high dose vs placebo and low dose vs placebo
- Relative change in tibia cortical area at 2 years and 1 year, high dose vs placebo and low dose vs placebo
- Relative change in tibia cortical vBMD at 2 years and 1 year, high dose vs placebo and low dose vs placebo

- Relative change in bone formation marker PN1P at 2 years and 1 year, high dose vs placebo and low dose vs placebo
- Relative change in bone resorption marker CTX at 2 years and 1 year, high dose vs placebo and low dose vs placebo
- Relative change in plasma butyrate, acetate, and propionate concentrations at 2 years and 1 year, high dose vs placebo and low dose vs placebo

The study's additional secondary variables that will be analyzed at a later stage are the following:

- Relative change in fecal calprotectin concentration at 2 years and 1 year, high dose vs placebo and low dose vs placebo
- Relative change in fecal lipocalin-2 concentration at 2 years and 1 year, high dose vs placebo and low dose vs placebo
- Relative change in serum Wnt10b concentration at 2 years and 1 year, high dose vs placebo and low dose vs placebo

4.5 Safety Variables

4.5.1 Exposure and Compliance

The exposure will be described as treatment duration in days, i.e. date for last dose taken – date for first dose taken + 1. The compliance, expressed as a percentage, both as continuous and in categories <80%, ≥80%, will be calculated as (total number of doses received-total number of doses returned)/(study duration in days*2)*100, where study duration is last date in study-first date in study+1. Both exposure and compliance will be described for the whole study.

4.5.2 Adverse Events

Treatment emergent adverse events (AE) are those that have started on or after the first dose of L. reuteri taken in the study. The AEs will be collected continuously during the study and will be coded to ICD-10 codes before summarized.

4.5.3 Quality of Life Variables

4.5.3.1 Gastrointestinal symptom rating scale

Gastrointestinal symptom rating scale (GSRS) will be described at baseline, 1 year and 2 years, for the total GSRS score and for the five sub-scales:

- Reflux-score
- Abdominal pain-score
- Indigestion-score

- Diarrhoea-score
- Constipation-score

5 Statistical Methods

5.1 Sample Size

The primary endpoint is 2 year relative change in tibia volumetric BMD measured with HRpQCT. The expected effects of supplementation are described in Table 2 below:

Variable	L.reuteri 6475 high dose (G1)	L.reuteri 6475 low dose (G2)	Placebo (G3)	Drop-out
Tibia total vBMD (ttvBMD) at baseline	230 ± 40	230 ± 40	230 ± 40	
% change in ttvBMD at one year (Secondary)	-0.90 ± 1.6	-1.08 ± 1.6	-1.85 ± 1.6	10%
% change in ttvBMD BMD at two years (Primary)	-1.75 ± 1.6	-2.10 ± 1.6	-3.50 ± 1.6	15%
SD = 1.6 (variance 2.56) is divided into 1.52 for between individuals' SD (variance 2.30) and 0.51 for within individual SD (variance 0.26). The total variance (0.26+2.30) agrees with the assumed 3.56 and ICC of ~0.90 (2.30/2.56) agrees with the observed in the first LR6475 study (ELBOW) performed on older women. In a similar way, SD of 2 is divided into 1.90 for between-subject SD and 0.63 for within subject SD.				

A smaller group to group difference is anticipated using the low-dose (G2) supplementation. For the power calculation, data was simulated according to the above table for 1000 studies. For each study, 10% and 15% missing data was assumed at 1 and 2 years, respectively. The analysis is performed with a mixed model for repeated measurements with time, group, time x group and baseline value as fixed effects and multiple imputation based on sampling of 50. Statistical power is based on 0.05 or 0.025 with Bonferroni-correction. The primary outcome (confirmed analysis) will be relative change in tibia total vBMD at 2 years. Using Bonferroni correction, the statistical power will be:

- G1 vs G3 results in power ≥99.9%
- G2 vs G3 results in power ≥99.9%

Secondary outcomes will be spine BMD, total hip BMD, tibia total volumetric BMD, cortical area, trabecular bone volume fraction, bone turnover markers (serum CTX and P1NP) at 2 years and at 1 year. At 1 year, total vBMD will also be a secondary analysis. For relative change in tibia total vBMD at 1 year the power is estimated to:

- G1 vs G3 results in power 95.0%
- G2 vs G3 results in power 81.9%

Assuming an alpha of 0.05.

For relative change in spine BMD at 2 years the power is estimated to:

- G1 vs G3 results in power 99.8%
- G2 vs G3 results in power 86.8%

In total, 72 to 80 women per treatment group were planned to be included in this study.

5.2 General Methodology

Categorical variables will be described by number and percentage and continuous variables by mean, standard deviation (SD), median, minimum and maximum.
There were 16/239 (6.7%) early discontinuations in this study. Due to the low number of drop-outs all analyses will be performed using mixed models for repeated measures (MMRM) where missing data is considered as missing completely at random, or complete case, as applicable. A sensitivity analysis of the primary variable will be performed using multiple imputation with 50 study samples including baseline patient characteristics and baseline value of the actual outcome variable included in the imputation regression model. Multiple imputation will be performed separately per each treatment group. All variables will be modelled in one and the same model using PROC MI procedure in SAS using fully conditional specification (FCS) with regression method (REG). The seed 73264 will be used. The outcome variables of relative change of the primary variable will be imputed. All baseline variables, medical history and prior medications will be included if they are associated with at least $r \geq 0.40$ to the primary outcome variable or absolute value of the primary variable at 2 years, or if baseline variables are associated to the missingness at 0.10 significance level. PROC MIANALYZE will be used for the purpose to pool results. Trace plots od means and standard deviations will be checked.

The difference between the treatment groups with respect to efficacy variables collected at baseline, 1 year and 2 years, that are all continuous, will be tested by using MMRM with relative change from baseline to 1 year and 2 years as dependent variable, treatment group, timepoint and interaction treatment group x timepoint as fixed effects, and baseline value as covariate. The optimal covariance pattern will be selected, e.g. compound symmetry, Toeplitz, autoregressive or unstructured homo- or heterogeneous for the three groups, by using the model with best goodness-of-fit value, the lowest Akaike Information Criterion (AIC). From this model Least Square Means (LSM) with 95% Confidence Intervals (CI) with p-values will be presented. The p-value for change from baseline within the two groups will be obtained from the same model. The normality and appropriateness to run regression models with normal distribution assumption will be checked for each outcome variable. In case this assumption is not satisfied an attempt to transform the variable to normal distribution will be made, and if this is not a successful approach non-parametric methods will be used, Mann-Whitney U-test for test between two groups and Wilcoxon Signed Rank test for test within one group.

For tests between two treatment groups Fisher's Exact test will be used for dichotomous variables, Mantel-Haenszel Chi-square trend test for ordered categorical variables, two-sample t-test for normally distributed continuous variables and Mann-Whitney U-test for other continuous variables. The relation between two normally distributed continuous variables will be described and tested by Pearson correlation coefficient in the case of normally distributed data, and otherwise by Spearman correlation coefficient.

All primary and secondary variables will be analyzed both for the ITT and PP population.

All tests will be two-tailed. All analyses will be performed by using SAS software version 9.4 (SAS Institute Inc., Cary, NC, USA).

5.3 Adjustment of Type I Error

Following rules will be applied for adjustment of Type I error:

1. Primary analysis: high dose vs placebo and low dose vs placebo evaluated at 0.025 significance level. If these two analyses are confirmed, then continue confirming following:
2. Confirmatory secondary analyses as per section 4.4.2 (11 tests):
    a. Use alpha 0.05 carried forward from the primary analysis above in case both primary variables were confirmed and apply Bonferroni-Holm adjustment on the 11 tests.
    b. Use alpha 0.025 carried forward from the primary analysis above in case only one primary variable was confirmed and apply Bonferroni-Holm adjustment on the 11 tests.
    c. Stop testing if none of the primary variables could be confirmed.

For the exploratory secondary analyses as per section 4.4.2 Bonferroni-Holm adjustment will be provided beside the unadjusted p-values, all conducted at 0.05 significance level.

5.4 Patient Disposition and Datasets Analyzed

The number of women included in each of the ITT, PP and safety populations will be summarized for each treatment group and overall. The number and percentage of women randomized and treated will be presented. Women who completed the study and those who withdrew from study prematurely will also be presented with a breakdown of the reasons for withdrawal by treatment group for the ITT, PP and safety populations.

5.5 Protocol Deviations

Major protocol deviations are those that are considered to have an impact on the analysis. The number of women with major protocol deviations will be summarized per treatment group.

5.6 Demographics and Baseline Characteristics

Demographics and baseline characteristics will be summarized by treatment group for the ITT and PP population and analyzed according to the methods described in section General Methodology above. High and low dose treatment groups will be compared to placebo group.

5.7 Medical History

Medical history will be summarized for each treatment group for the ITT population.

5.8 Prior Medications

Prior medications will be summarized for each treatment group for the ITT population.

5.9 Efficacy Analyses

5.9.1 Primary Analysis

The primary analysis in this study is to test the difference between the *L. reuteri* high dose vs Placebo and *L. reuteri* low dose vs Placebo with respect to relative change from baseline to 2 years in total tibia vBMD (%), performed by using MMRM adjusted for total tibia vBMD baseline value.

The main primary analysis will be performed on the ITT population using MMRM as described in the General Methodology above.

Sensitivity analysis 1 of the primary variable will be performed by adopting multiple imputation for early discontinued women as per General Methodology above.

Sensitivity analysis 2 of the primary variable will be done by adding baseline confounders, i.e. variables that are statistically significantly related to the outcome and that are differing statistically significantly between any pair of the treatment groups, into the model on the ITT population.

The primary variable will also be analysed for the PP population.
Actual values, change from baseline and relative change from baseline will be described for study visits.
The raw data for total tibia vBMD at baseline, at 1 year and at 2 years will be presented as boxplots per treatment group.

5.9.2 Secondary Analyses

The secondary analyses will be performed as defined in the General Methodology above on the ITT and the PP population. Actual values, change from baseline and relative change from baseline will be described for study visits. All raw data for variables over time will be shown as boxplots per treatment group.

5.10 Subgroup Analyses

The primary and all secondary efficacy variables will be evaluated for following subgroups (defined at baseline) on the ITT population:

- Years since menopause (<median years, ≥median years)
- BMI (<25 kg/m^2, ≥25 kg/m^2)
- IPAQ (<median METs/week, ≥median METs/week)

5.11 Safety Analyses

5.11.1 Exposure and Compliance

Exposure and compliance will be summarized for each treatment group overall for the whole study. The summaries will be provided for the safety population.

5.11.2 Adverse Events

Only treatment-emergent AEs will be included in the summaries for the safety population. A summary of subjects reporting at least one of the following AEs will be presented in an overview table:

- Any AE
- Any SAE
- Any treatment-related AE
- Any treatment-related SAE
- Any AE leading to discontinuation
- Any fractures
- Death

Summaries per CTCAE (v5) presenting n (%) of AEs and n (%) of subjects with at least one AE will be provided for:

- All AEs (includes all serious and non-serious AEs)
- All AEs by maximum reported intensity
- All AEs by causality
- All SAEs
- All AEs leading to discontinuation

5.11.3 Quality of Life

QoL variables will be described over time per treatment group for the safety population.

6 Interim Analyses

None.

7 Changes from Study Protocol

I. Definition of the per protocol population.

The new definition of the per protocol population: Women who during the study use oral glucocorticoids for more than 2 weeks, use osteoporosis medication (bisphosphonates, denosumab or teriparatide), menopausal hormone therapy, or consume other probiotic supplements will be excluded from the per protocol analysis.

II. Additional secondary outcomes to be analyzed

Secondary objectives are relative change in after 24 (secondary first) and 12 (secondary last) months in

- Plasma acetate
- Plasma propionate

III. Change in biochemical analyses as per

- Butyrate will be analyzed in plasma, not in serum
- CTX will be analyzed in plasma, not in serum

Serum Wnt10b will be analyzed at a later stage, when appropriate methods are available.

Fecal calprotectin and lipocalin-2 will be analyzed at a later stage and will not be part of the primary analyses.

8 Planned Tables and Figures

The number and order of the tables and figures might change as appropriate.

Table Number	Table Title
**Table 1.1**	Patient disposition and data sets analyzed (ITT population)
**Table 1.2**	Protocol violation leading to exclusion from PP population (ITT population)
**Table 2.1**	Demographics and baseline characteristics (ITT population)
**Table 2.2**	Demographics and baseline characteristics (PP population)
**Table 3.1**	Medical history (ITT population)
**Table 3.2**	Prior medications (ITT population)
**Table 4**	Analyses for identification of potential confounders (ITT population)
**Table 5.1**	Main and sensitivity analyses of the primary efficacy variable (ITT population)
**Table 5.2**	Analyses of the primary efficacy variable (PP population)
**Table 6.1**	Confirmatory analyses of the secondary efficacy variables (ITT population)
**Table 6.2**	Confirmatory analyses of the secondary efficacy variables (PP population)
**Table 7.1**	Exploratory analyses of the secondary efficacy variables (ITT population)
**Table 7.2**	Exploratory analyses of the secondary efficacy variables (PP population)
**Table 8**	Correlation analyses between primary, secondary variables and continuous age, BMI and IPAQ (ITT population)
**Table 9.1**	Subgroup analyses of the primary and secondary variables by age group (ITT population)
**Table 9.2**	Subgroup analyses of the primary and secondary variables by BMI group (ITT population)
**Table 9.3**	Subgroup analyses of the primary and secondary variables by IPAQ group (ITT population)
**Table 10**	Exposure and compliance (Safety population)
**Table 11.1**	Summary of adverse events (Safety population)
**Table 11.2**	Adverse events by ICD-10 code (Safety population)
**Table 11.3**	Adverse events by ICD-10 code and maximum severity (Safety population)
**Table 11.4**	Adverse events by ICD-10 code and causality assessment (Safety population)
**Table 11.5**	Serious adverse events by ICD-10 code (Safety population)
**Table 11.6**	Adverse events leading to discontinuation by ICD-10 code (Safety population)
**Table 12**	Descriptive data for GSRS over time (Safety population)

Figure Number	Figure Title
**Figure x.1**	Boxplots for relative change from baseline to 1 year and 2 years for xxxx per treatment group (ITT population)

**Figure x.2**	Boxplots for actual values at baseline, 1 year and 2 years for xxxx per treatment group (ITT population)
**Figure x.1**	Scatter plots for age at baseline vs xxx (ITT population)
**Figure x.2**	Scatter plots for BMI at baseline vs xxx (ITT population)
**Figure x.3**	Scatter plots for IPAQ at baseline vs xxx (ITT population)
**Figure x.1**	Forest plot for subgroup analyses of primary and secondary variables by age category (ITT population)
**Figure x.2**	Forest plot for subgroup analyses of primary and secondary variables by BMI category (ITT population)
**Figure x.3**	Forest plot for subgroup analyses of primary and secondary variables by IPAQ category (ITT population)
