# Supplementary material for: Limosilactobacillus reuteri 6475 and Prevention of Early Postmenopausal Bone Loss: A Randomized Clinical Trial
Source: JAMA Netw Open. 2024 Jun 12;7(6):e2415455. doi: 10.1001/jamanetworkopen.2024.15455 (PMC11170297; doi:10.1001/jamanetworkopen.2024.15455)
Supplement: Supplement 3. — eMethods. Extended Methods - Blood Biochemistry Analysis and Statistical Methods eReference eTable 1. Patient Disposition and Data Sets Analyzed (ITT Population) eTable 2. Medical History (ITT Population) eTable 3. Prior Medications (ITT Population) eTable 4. Change in the Tibia Total Volumetric BMD According to Treatment Using Multiple Imputation in the ITT Population eTable 5. Main and Sensitivity Analyses of the Percent Change in Bone Characteristics and Bone Turnover Markers in the PP Population eTable 6. Compliance (Safety Population) eTable 7. Adverse Events by System Organ Class and per Treatment in the Safety Population eTable 8. Analyses of the GSRS in the Safety Population eTable 9. Exploratory Analyses of the Change in Serum Short-Chain Fatty Acids in the ITT Population eTable 10. Subgroup Analyses of the Primary Efficacy Variable (ITT Population) eTable 11. Logistic Regression for Responders Based on the Responders Defined as Percent Change in Total Tibia vBMD at Two Years>-1.0 (All High- and Low-Dose Participants Included) eFigure. Pearson Correlation Between BMI and % Change in Tibia Total vBMD by Treatment Group Over Time (ITT Population) [file jamanetwopen-e2415455-s003.pdf]

Supplementary Online Content

Gregori G, Pivodic A, Magnusson P, et al. *Limosilactobacillus reuteri* 6475 and prevention of early postmenopausal bone loss: a randomized clinical trial. *JAMA Netw Open*. 2024;7(6):e2415455. doi:10.1001/jamanetworkopen.2024.15455

eMethods. Extended Methods - Blood Biochemistry Analysis and Statistical Methods

eReference

eTable 1. Patient Disposition and Data Sets Analyzed (ITT Population)

eTable 2. Medical History (ITT Population)

eTable 3. Prior Medications (ITT Population)

eTable 4. Change in the Tibia Total Volumetric BMD According to Treatment Using Multiple Imputation in the ITT Population

eTable 5. Main and Sensitivity Analyses of the Percent Change in Bone Characteristics and Bone Turnover Markers in the PP Population

eTable 6. Compliance (Safety Population)

eTable 7. Adverse Events by System Organ Class and per Treatment in the Safety Population

eTable 8. Analyses of the GSRS in the Safety Population

eTable 9. Exploratory Analyses of the Change in Serum Short-Chain Fatty Acids in the ITT Population

eTable 10. Subgroup Analyses of the Primary Efficacy Variable (ITT Population)

eTable 11. Logistic Regression for Responders Based on the responders Defined as Percent Change in Total Tibia vBMD at Two Years >-1.0 (All High- and Low-Dose Participants Included)

eFigure. Pearson Correlation Between BMI and % Change in Tibia Total vBMD by Treatment Group Over Time (ITT Population)

This supplementary material has been provided by the authors to give readers additional information about their work.

© 2024 Gregori G et al. *JAMA Network Open.*

eMethods - Extended methods - Blood biochemistry analysis and statistical methods.

Procedures

The study participants were randomized between December 4th 2019 and September 30th 2020. The last study participant visit was on October 6th 2022.

Blood biochemistry

Fasting morning blood samples were collected from all study participants. Aliquots of serum and plasma were stored at –80°C until analysis. Serum type I procollagen intact N-terminal propeptide (PINP) was assessed with the UniQ radioimmunoassay (Aidian Oy, Espoo, Finland), with an assay performance of: analytical range 5–250 µg/L, intra-assay CV of <5%, and interassay CV of <6%. C-terminal telopeptide cross-links of collagen type I (CTX) was measured in EDTA plasma samples by the CrossLaps® enzyme-linked immunosorbent assay (Immunodiagnostic Systems Holdings PLC, Boldon, UK), with an assay performance of: analytical range 0.02–3.38 ng/mL, intra-assay CV of <6%, and interassay CV of <10%.

Serum 25-OH-vitamin D and calcium were measured at a Swedac accredited clinical chemistry laboratory (accreditation number 1240) at the Sahlgrenska University Hospital. Serum 25-OH-vitamin D was measured from blood samples with Alinity i, a chemiluminescent

microparticle immunoassay (CMIA). The CVs were 8% and 6% at concentrations 25 nmol/l and 45 nmol/l, respectively. Calcium was measured in plasma samples using Cobas 6000, with CVs of 3% and 4% at concentrations of 2 mmol/l and 3mmol/l, respectively. Circulating levels of short chain fatty acids (SCFA) butyrate, acetate, and propionate were analyzed in EDTA plasma by liquid chromatography–mass spectrometry (LC-MS) according to a method described previously,[1] with some modifications, at the Department of Biology and Biological Engineering, Chalmers University of Technology, Gothenburg.

Statistical methods

Categorical variables are described by number and percentage and continuous variables by mean and standard deviation (SD). There were 16/239 (6.7%) early discontinuations during follow-up. The differences between the treatment groups with respect to the efficacy variables collected at baseline, 1 year, and 2 years, which are all continuous, were tested by using mixed models for repeated measurements (MMRM) with relative change from baseline to 1 year and 2 years as the dependent variable, treatment group, timepoint, and interaction treatment group x timepoint as fixed effects, and baseline value as a covariate. This method handles missing data as being completely at random. An unstructured covariance pattern was used for correlated data repeated over time for the three groups. From this model, Least Square Means (LSM) with 95% Confidence Intervals (CI) and p-values were presented. The normality and appropriateness to run regression models with normal distribution assumption were checked for each outcome variable and the assumption was satisfied.

A sensitivity analysis of the primary endpoint employed the multiple imputation technique. In total 12 (5.0%) had missing primary endpoint at 1 year, and 16 (6.7%) at 2 years. Baseline patient characteristics and the baseline value of the primary outcome variable were included in the imputation regression model (SAP).

Additional exploratory subgroup analyses, that were not predefined and described in the SAP, were performed using logistic regression, to investigate which baseline variables were associated with a positive treatment response (defined as a percent change in total vBMD at 2 years of >-1).

For tests between two treatment groups, Fisher's Exact test was used for dichotomous variables, Mantel-Haenszel Chi-square trend test for ordered categorical variables, two-sample t-test for normally distributed continuous variables, and Mann-Whitney U-test for other continuous variables. The relation between two normally distributed continuous variables was described and tested by Pearson correlation coefficient in the case of normally distributed data, and otherwise by Spearman correlation coefficient. All primary and secondary variables were analyzed both for the ITT and PP population. All tests were two-tailed. To account for type 1 error, a pre-planned alpha level of 0.025 was used for the two active comparisons against the placebo with respect to the primary endpoint. Bonferroni-Holm adjustment was planned to be applied to the secondary endpoints. All analyses were performed by using SAS software version 9.4 (SAS Institute Inc., Cary, NC, USA).

© 2024 Gregori G et al. *JAMA Network Open.*

eReference

1. Han J, Lin K, Sequeira C, Borchers CH. An isotope-labeled chemical derivatization method for the quantitation of short-chain fatty acids in human feces by liquid chromatography-tandem mass spectrometry. *Anal Chim Acta*. Jan 7 2015;854:86-94. doi:10.1016/j.aca.2014.11.015

© 2024 Gregori G et al. *JAMA Network Open.*

eTable 1. Patient disposition and data sets analyzed (ITT population).

Variable	High-dose N=80	Low-dose N=80	Placebo N=79
ITT population	80 (100.0%)	80 (100.0%)	79 (100.0%)
Safety population	80 (100.0%)	80 (100.0%)	79 (100.0%)
PP population	62 (77.5%)	66 (82.5%)	65 (82.3%)
Reason for exclusion from PP population			
Use of glucocorticoids	1 (5.6%)	0 (0.0%)	1 (7.1%)
Use of menopausal hormonal treatment	4 (22.2%)	1 (7.1%)	3 (21.4%)
Early discontinuation	5 (27.8%)	6 (42.9%)	5 (35.7%)
Poor compliance (<80%)	8 (44.4%)	7 (50.0%)	5 (35.7%)
Early discontinuation	5 (6.3%)	6 (7.5%)	5 (6.3%)
Reason for discontinuation			
Withdrawn consent	2 (40.0%)	5 (83.3%)	2 (40.0%)
AE	0 (0.0%)	0 (0.0%)	2 (40.0%)
Lost-to follow-up	3 (60.0%)	1 (16.7%)	1 (20.0%)

Data are presented as number of observations, or number (percentage).

© 2024 Gregori G et al. *JAMA Network Open.*

eTable 2. Medical history (ITT population).

Variable	High-dose N=80	Low-dose N=80	Placebo N=79
Diabetes	0 (0.0%)	0 (0.0%)	0 (0.0%)
Hyperthyroidism	0 (0.0%)	0 (0.0%)	0 (0.0%)
Hypothyroidism	8 (10.0%)	5 (6.3%)	15 (19.0%)
Osteoporosis	0 (0.0%)	0 (0.0%)	0 (0.0%)
Chronic bronchitis, asthma or emphysema	0 (0.0%)	4 (5.0%)	7 (8.9%)
Stroke	0 (0.0%)	0 (0.0%)	1 (1.3%)
Hypertension	10 (12.5%)	6 (7.5%)	5 (6.3%)
Myocardial infarction	0 (0.0%)	0 (0.0%)	0 (0.0%)
Angina	0 (0.0%)	0 (0.0%)	0 (0.0%)
Congestive heart disease	0 (0.0%)	1 (1.3%)	0 (0.0%)
Chronic liver disease	0 (0.0%)	0 (0.0%)	0 (0.0%)
Osteoarthritis	8 (10.0%)	15 (18.8%)	15 (19.0%)
Gout	0 (0.0%)	0 (0.0%)	0 (0.0%)
Muscle rheumatism	0 (0.0%)	0 (0.0%)	1 (1.3%)
Cancer	2 (2.5%)	5 (6.3%)	1 (1.3%)
Prevalent falls (during the last year)	15 (18.8%)	14 (17.5%)	17 (21.5%)
Prevalent fracture	9 (11.3%)	21 (26.3%)	11 (13.9%)

Data are presented as number (percentage).

© 2024 Gregori G et al. *JAMA Network Open.*

eTable 3. Prior medications (ITT population).

Variable	High-dose N=80	Low-dose N=80	Placebo N=79
Hypothyroidism treatment	8 (10.0%)	5 (6.3%)	15 (19.0%)
Osteoporosis treatment	0 (0.0%)	0 (0.0%)	0 (0.0%)
Hypertension treatment	9 (11.3%)	3 (3.8%)	2 (2.5%)
Angina treatment	0 (0.0%)	0 (0.0%)	0 (0.0%)
Congestive heart disease treatment	0 (0.0%)	0 (0.0%)	0 (0.0%)
Chronic bronchitis, asthma or emphysema treatment	0 (0.0%)	2 (2.5%)	5 (6.3%)
Chronic liver disease treatment	0 (0.0%)	0 (0.0%)	0 (0.0%)
Muscle rhemautism (oral GC treatment)	0 (0.0%)	0 (0.0%)	1 (1.3%)
Arthritis or joint pain medication	4 (5.1%)	2 (2.5%)	1 (1.3%)
Missing	1	0	0
Calcium and vitamin D supplements	16 (20.3%)	13 (16.5%)	12 (15.2%)
Missing	1	1	0

Data are presented as number (percentage). GC= oral glucocorticoid treatment.

© 2024 Gregori G et al. *JAMA Network Open.*

eTable 4. Change in the tibia total volumetric BMD according to treatment using multiple imputation in the ITT population.

		LS means (95% CI) p-value			Difference in LS means (95% CI) p-value	
**Percent change (%)**	**Visit**	**High-dose**	**Low-dose**	**Placebo**	**High-dose - Placebo**	**Low-dose - Placebo**
**Total volumetric BMD**	**Year 1**	-1.20 (-1.64; -0.76) p<.0001	-1.04 (-1.47; -0.61) p<.0001	-1.20 (-1.63; -0.77) p<.0001	-0.00 (-0.62; 0.61) p=1.00	0.16 (-0.45; 0.77) p=0.61
	**Year 2**	-2.40 (-2.95; -1.85) p<.0001	-2.52 (-3.07; -1.97) p<.0001	-2.33 (-2.87; -1.78) p<.0001	-0.07 (-0.84; 0.70) p=0.86	-0.19 (-0.96; 0.57) p=0.62

Least Square Means (LSM) with 95% Confidence Intervals (CI) and p-values over time within groups and between groups are shown Mixed models for repeated measures are applied with percent difference as outcome variable, visit, treatment group, interaction visit*treatment group as main fixed effects and baseline value as covariate. Unstructured covariance pattern is used for correlated data repeated over time. Diagnostic plots of residuals were investigated and found satisfactory. Missing data is imputed using multiple imputation with 50 samplings. Variables used for imputation are: weight, BMI, body fat, appendicular lean mass,serum 25-OH-vitamin D, salt intake, baseline tibia ultradistal (standard site) total volumetric BMD, and baseline tibia ultradistal (standard site) trabecular bone volume fraction. Percent change at 24 months % change at 12 months was also used for imputation.

© 2024 Gregori G et al. *JAMA Network Open.*

eTable 5. Main and sensitivity analyses of the percent change in bone characteristics and bone turnover markers in the PP population.

		LS means (95% CI) p-value			Difference in LS means (95% CI) p-value	
**Percent change (%)**	**Visit**	**High-dose**	**Low-dose**	**Placebo**	**High-dose - Placebo**	**Low-dose - Placebo**
**Tibia total volumetric BMD**	**Year 1**	-1.24 (-1.73; -0.75) p<.0001	-0.92 (-1.39; -0.45) p=0.0002	-1.33 (-1.81; -0.86) p<.0001	0.09 (-0.59; 0.77) p=0.79	0.41 (-0.26; 1.08) p=0.23
	**Year 2**	-2.52 (-3.10; -1.94) p<.0001	-2.39 (-2.95; -1.82) p<.0001	-2.51 (-3.08; -1.95) p<.0001	-0.00 (-0.82; 0.81) p=0.99	0.13 (-0.68; 0.93) p=0.76
**Tibia trabecular bone volume fraction**	**Year 1**	-0.11 (-0.65; 0.44) p=0.70	0.03 (-0.50; 0.56) p=0.92	-0.49 (-1.02; 0.04) p=0.07	0.38 (-0.38; 1.15) p=0.33	0.52 (-0.24; 1.27) p=0.18
	**Year 2**	-0.72 (-1.37; -0.07) p=0.029	-0.94 (-1.57; -0.31) p=0.0035	-1.30 (-1.93; -0.66) p<.0001	0.58 (-0.33; 1.48) p=0.21	0.36 (-0.53; 1.25) p=0.43
**Tibia cortical area**	**Year 1**	-2.43 (-3.18; -1.68) p<.0001	-1.95 (-2.67; -1.22) p<.0001	-2.11 (-2.84; -1.38) p<.0001	-0.32 (-1.37; 0.73) p=0.55	0.17 (-0.86; 1.19) p=0.75
	**Year 2**	-4.02 (-4.97; -3.07) p<.0001	-3.82 (-4.74; -2.90) p<.0001	-3.39 (-4.31; -2.46) p<.0001	-0.63 (-1.97; 0.70) p=0.35	-0.43 (-1.74; 0.87) p=0.51
**Tibia cortical volumetric BMD**	**Year 1**	-0.94 (-1.23; -0.65) p<.0001	-0.71 (-0.99; -0.43) p<.0001	-0.83 (-1.11; -0.55) p<.0001	-0.11 (-0.51; 0.29) p=0.59	0.12 (-0.27; 0.52) p=0.55
	**Year 2**	-1.93 (-2.28; -1.58) p<.0001	-1.63 (-1.96; -1.29) p<.0001	-1.63 (-1.97; -1.29) p<.0001	-0.30 (-0.79; 0.18) p=0.22	0.01 (-0.47; 0.48) p=0.98
**Bone mineral density, lumbar spine**	**Year 1**	-1.31 (-1.95; -0.68) p<.0001	-1.86 (-2.47; -1.24) p<.0001	-1.25 (-1.88; -0.63) p=0.0001	-0.06 (-0.95; 0.83) p=0.89	-0.60 (-1.48; 0.27) p=0.18
	**Year 2**	-2.10 (-2.82; -1.39) p<.0001	-2.26 (-2.95; -1.56) p<.0001	-2.26 (-2.96; -1.56) p<.0001	0.16 (-0.84; 1.16) p=0.76	0.00 (-0.98; 0.99) p=0.99
**Bone mineral density, total hip**	**Year 1**	-1.65 (-2.20; -1.11) p<.0001	-1.44 (-1.97; -0.91) p<.0001	-1.30 (-1.84; -0.77) p<.0001	-0.35 (-1.12; 0.41) p=0.37	-0.14 (-0.90; 0.61) p=0.71
	**Year 2**	-2.63 (-3.25; -2.00) p<.0001	-2.32 (-2.93; -1.72) p<.0001	-2.38 (-2.99; -1.77) p<.0001	-0.25 (-1.12; 0.63) p=0.58	0.06 (-0.80; 0.92) p=0.90
**CTX**	**Year 1**	11.37 (1.25; 21.50) p=0.028	13.19 (3.54; 22.85) p=0.0077	3.58 (-6.11; 13.27) p=0.47	7.79 (-6.23; 21.81) p=0.27	9.61 (-4.07; 23.29) p=0.17
	**Year 2**	2.19 (-7.42; 11.79) p=0.65	6.90 (-2.31; 16.12) p=0.14	10.43 (1.29; 19.58) p=0.026	-8.25 (-21.52; 5.02) p=0.22	-3.53 (-16.52; 9.46) p=0.59
**PINP**	**Year 1**	9.12 (1.20; 17.04) p=0.024	1.40 (-6.30; 9.10) p=0.72	-3.74 (-11.44; 3.95) p=0.34	12.86 (1.79; 23.93) p=0.023	5.14 (-5.73; 16.02) p=0.35
	**Year 2**	2.35 (-7.34; 12.04) p=0.63	-1.17 (-10.61; 8.27) p=0.81	-9.58 (-19.02; -0.14) p=0.047	11.93 (-1.62; 25.48) p=0.08	8.41 (-4.92; 21.74) p=0.21

© 2024 Gregori G et al. *JAMA Network Open.*

		LS means (95% CI)<br>p-value			Difference in LS means (95% CI)<br>p-value	
**Percent change (%)**	**Visit**	**High-dose**	**Low-dose**	**Placebo**	**High-dose - Placebo**	**Low-dose - Placebo**

Least Square Means (LSM) with 95% Confidence Intervals (CI) and p-values over time within groups and between groups are shown. Mixed models for repeated measures are applied with percent difference as outcome variable, visit, treatment group, interaction visit*treatment group as main fixed effects and baseline value as covariate. Tibia results are from the ultradistal (standard site) measurement.

© 2024 Gregori G et al. *JAMA Network Open.*

eTable 6. Compliance (Safety population).

Variable	High-dose N=80	Low-dose N=80	Placebo N=79
Compliance (%)	87.5±24.6<br>96.5 (0.5 - 99.8)<br>n=75	89.1±21.6<br>96.6 (0.9 - 100.7)<br>n=74	93.6±12.9<br>97.3 (12.6 - 102.1)<br>n=74
Compliance >=80%			
No	15 (18.8%)	13 (16.3%)	10 (12.7%)
Yes	65 (81.3%)	67 (83.8%)	69 (87.3%)

Data are presented as mean±standard deviation, median (range) and number of observations, or number (percentage).

© 2024 Gregori G et al. *JAMA Network Open.*

eTable 7. Adverse events by system organ class and per treatment in the safety population.

	High-dose N=80		Low-dose N=80		Placebo N=79	
	n Events	n (%) women with events	n Events	n (%) women with events	n Events	n (%) women with events
**Any AE**	**338**	**80 (100.0%)**	**322**	**74 (92.5%)**	**408**	**77 (96.3%)**
**Blood and lymphatic system disorders**	**0**	**0 (0.0%)**	**1**	**1 (1.3%)**	**0**	**0 (0.0%)**
Anemia	0	0 (0.0%)	1	1 (1.3%)	0	0 (0.0%)
**Cardiac disorders**	**0**	**0 (0.0%)**	**2**	**2 (2.5%)**	**1**	**1 (1.3%)**
Chest pain - cardiac	0	0 (0.0%)	1	1 (1.3%)	0	0 (0.0%)
Palpitations	0	0 (0.0%)	1	1 (1.3%)	1	1 (1.3%)
**Ear and labyrinth disorders**	**1**	**1 (1.3%)**	**8**	**7 (8.8%)**	**8**	**5 (6.3%)**
Ear pain	0	0 (0.0%)	1	1 (1.3%)	0	0 (0.0%)
External ear pain	0	0 (0.0%)	1	1 (1.3%)	0	0 (0.0%)
Tinnitus	0	0 (0.0%)	1	1 (1.3%)	0	0 (0.0%)
Vertigo	1	1 (1.3%)	5	4 (5.0%)	8	5 (6.3%)
**Eye disorders**	**1**	**1 (1.3%)**	**1**	**1 (1.3%)**	**8**	**7 (8.9%)**
Cataract	0	0 (0.0%)	0	0 (0.0%)	1	1 (1.3%)
Eye disorders - allergy	1	1 (1.3%)	0	0 (0.0%)	0	0 (0.0%)
Eye disorders - increased pressure	0	0 (0.0%)	1	1 (1.3%)	2	2 (2.5%)
Eye disorders - inflammation	0	0 (0.0%)	0	0 (0.0%)	1	1 (1.3%)
Scleral disorder	0	0 (0.0%)	0	0 (0.0%)	1	1 (1.3%)
Vision decreased	0	0 (0.0%)	0	0 (0.0%)	3	3 (3.8%)
**Gastrointestinal disorders**	**85**	**49 (61.3%)**	**70**	**42 (52.5%)**	**102**	**54 (68.4%)**
Abdominal pain	5	5 (6.3%)	7	6 (7.5%)	15	13 (16.5%)
Anal hemorrhage	1	1 (1.3%)	1	1 (1.3%)	0	0 (0.0%)
Bloating	18	16 (20.0%)	17	17 (21.3%)	18	18 (22.8%)
Constipation	9	8 (10.0%)	10	6 (7.5%)	9	8 (10.1%)
Diarrhea	14	9 (11.3%)	5	5 (6.3%)	12	10 (12.7%)
Dry mouth	2	1 (1.3%)	3	2 (2.5%)	1	1 (1.3%)
Dyspepsia	0	0 (0.0%)	0	0 (0.0%)	1	1 (1.3%)
Dysphagia	1	1 (1.3%)	0	0 (0.0%)	0	0 (0.0%)
Flatulence	10	10 (12.5%)	8	6 (7.5%)	11	9 (11.4%)
Gastroesophageal reflux disease	9	8 (10.0%)	8	5 (6.3%)	12	9 (11.4%)
Gastrointestinal disorders - more frequent defacation	1	1 (1.3%)	1	1 (1.3%)	1	1 (1.3%)
Nausea	5	5 (6.3%)	4	3 (3.8%)	10	8 (10.1%)
Small intestinal mucositis	3	3 (3.8%)	1	1 (1.3%)	2	1 (1.3%)
Stomach pain	1	1 (1.3%)	0	0 (0.0%)	1	1 (1.3%)
Toothache	4	3 (3.8%)	2	2 (2.5%)	6	4 (5.1%)
Vomiting	2	2 (2.5%)	3	3 (3.8%)	3	3 (3.8%)
**General disorders and administration site conditions**	**26**	**17 (21.3%)**	**22**	**19 (23.8%)**	**26**	**18 (22.8%)**
Edema face	1	1 (1.3%)	0	0 (0.0%)	0	0 (0.0%)
Facial pain	1	1 (1.3%)	0	0 (0.0%)	0	0 (0.0%)
Fatigue	0	0 (0.0%)	1	1 (1.3%)	0	0 (0.0%)
Fever	0	0 (0.0%)	2	2 (2.5%)	0	0 (0.0%)
Flu like symptoms	21	13 (16.3%)	14	13 (16.3%)	20	16 (20.3%)
Malaise	2	2 (2.5%)	5	5 (6.3%)	5	3 (3.8%)
Non-cardiac chest pain	0	0 (0.0%)	0	0 (0.0%)	1	1 (1.3%)
Pain	1	1 (1.3%)	0	0 (0.0%)	0	0 (0.0%)

© 2024 Gregori G et al. *JAMA Network Open.*

	High-dose N=80		Low-dose N=80		Placebo N=79	
	n Events	n (%) women with events	n Events	n (%) women with events	n Events	n (%) women with events
**Hepatobiliary disorders**	**0**	**0 (0.0%)**	**0**	**0 (0.0%)**	**1**	**1 (1.3%)**
Gallbladder obstruction	0	0 (0.0%)	0	0 (0.0%)	1	1 (1.3%)
**Immune system disorders**	**2**	**2 (2.5%)**	**3**	**3 (3.8%)**	**0**	**0 (0.0%)**
Allergic reaction	2	2 (2.5%)	3	3 (3.8%)	0	0 (0.0%)
**Infections and infestations**	**91**	**53 (66.3%)**	**76**	**45 (56.3%)**	**104**	**51 (64.6%)**
Bronchial infection	0	0 (0.0%)	0	0 (0.0%)	1	1 (1.3%)
Eye infection	0	0 (0.0%)	3	1 (1.3%)	2	2 (2.5%)
Infections and infestations - Other COVID-19	30	27 (33.8%)	32	29 (36.3%)	27	25 (31.6%)
Kidney infection	1	1 (1.3%)	0	0 (0.0%)	0	0 (0.0%)
Laryngitis	0	0 (0.0%)	0	0 (0.0%)	1	1 (1.3%)
Lung infection	1	1 (1.3%)	0	0 (0.0%)	0	0 (0.0%)
Otitis externa	1	1 (1.3%)	0	0 (0.0%)	0	0 (0.0%)
Otitis media	0	0 (0.0%)	0	0 (0.0%)	1	1 (1.3%)
Shingles	1	1 (1.3%)	0	0 (0.0%)	3	3 (3.8%)
Skin infection	9	8 (10.0%)	3	3 (3.8%)	18	14 (17.7%)
Upper respiratory infection	32	23 (28.8%)	31	23 (28.8%)	40	26 (32.9%)
Urinary tract infection	16	10 (12.5%)	7	4 (5.0%)	10	6 (7.6%)
Vaginal infection	0	0 (0.0%)	0	0 (0.0%)	1	1 (1.3%)
**Injury, poisoning and procedural complications**	**8**	**8 (10.0%)**	**15**	**9 (11.3%)**	**16**	**12 (15.2%)**
Bruising	0	0 (0.0%)	3	3 (3.8%)	4	4 (5.1%)
Fall	5	5 (6.3%)	9	7 (8.8%)	6	6 (7.6%)
Fracture	3	3 (3.8%)	2	2 (2.5%)	3	3 (3.8%)
Hip fracture	0	0 (0.0%)	0	0 (0.0%)	1	1 (1.3%)
Spinal fracture	0	0 (0.0%)	1	1 (1.3%)	1	1 (1.3%)
Vertigo	0	0 (0.0%)	0	0 (0.0%)	1	1 (1.3%)
**Investigations**	**6**	**5 (6.3%)**	**2**	**2 (2.5%)**	**4**	**4 (5.1%)**
Weight gain	0	0 (0.0%)	1	1 (1.3%)	2	2 (2.5%)
Weight loss	6	5 (6.3%)	1	1 (1.3%)	2	2 (2.5%)
**Metabolism and nutrition disorders**	**0**	**0 (0.0%)**	**1**	**1 (1.3%)**	**1**	**1 (1.3%)**
Hyperglycemia	0	0 (0.0%)	1	1 (1.3%)	1	1 (1.3%)
**Musculoskeletal and connective tissue disorders**	**60**	**36 (45.0%)**	**67**	**38 (47.5%)**	**73**	**41 (51.9%)**
Arthralgia	13	10 (12.5%)	8	7 (8.8%)	11	9 (11.4%)
Arthritis	8	5 (6.3%)	8	7 (8.8%)	7	7 (8.9%)
Back pain	8	7 (8.8%)	14	12 (15.0%)	12	10 (12.7%)
Chest wall pain	0	0 (0.0%)	1	1 (1.3%)	1	1 (1.3%)
Muscle cramp	5	5 (6.3%)	0	0 (0.0%)	1	1 (1.3%)
Myalgia	0	0 (0.0%)	0	0 (0.0%)	2	2 (2.5%)
Neck pain	1	1 (1.3%)	1	1 (1.3%)	0	0 (0.0%)
Pain in extremity	25	21 (26.3%)	35	24 (30.0%)	39	25 (31.6%)
**Neoplasms benign, malignant and unspecified (incl cysts and polyps)**	**1**	**1 (1.3%)**	**0**	**0 (0.0%)**	**2**	**2 (2.5%)**
**Nervous system disorders**	**11**	**7 (8.8%)**	**13**	**13 (16.3%)**	**4**	**2 (2.5%)**
Headache	10	6 (7.5%)	10	10 (12.5%)	4	2 (2.5%)
Peripheral sensory neuropathy	1	1 (1.3%)	2	2 (2.5%)	0	0 (0.0%)
Tremor	0	0 (0.0%)	1	1 (1.3%)	0	0 (0.0%)
**Psychiatric disorders**	**6**	**5 (6.3%)**	**3**	**2 (2.5%)**	**7**	**3 (3.8%)**
Anxiety	0	0 (0.0%)	0	0 (0.0%)	2	1 (1.3%)
Depression	1	1 (1.3%)	0	0 (0.0%)	1	1 (1.3%)
Insomnia	5	4 (5.0%)	3	2 (2.5%)	4	3 (3.8%)

© 2024 Gregori G et al. *JAMA Network Open.*

	High-dose N=80		Low-dose N=80		Placebo N=79	
	n Events	n (%) women with events	n Events	n (%) women with events	n Events	n (%) women with events
**Reproductive system and breast disorders**	**1**	**1 (1.3%)**	**1**	**1 (1.3%)**	**3**	**3 (3.8%)**
Vaginal hemorrhage	1	1 (1.3%)	1	1 (1.3%)	3	3 (3.8%)
**Respiratory, thoracic and mediastinal disorders**	**11**	**8 (10.0%)**	**8**	**7 (8.8%)**	**20**	**16 (20.3%)**
Allergic rhinitis	9	6 (7.5%)	8	7 (8.8%)	14	11 (13.9%)
Cough	0	0 (0.0%)	0	0 (0.0%)	1	1 (1.3%)
Dyspnea	1	1 (1.3%)	0	0 (0.0%)	0	0 (0.0%)
Epistaxis	0	0 (0.0%)	0	0 (0.0%)	3	2 (2.5%)
Sleep apnea	1	1 (1.3%)	0	0 (0.0%)	1	1 (1.3%)
Sore throat	0	0 (0.0%)	0	0 (0.0%)	1	1 (1.3%)
**Skin and subcutaneous tissue disorders**	**16**	**15 (18.8%)**	**10**	**9 (11.3%)**	**15**	**11 (13.9%)**
Alopecia	0	0 (0.0%)	1	1 (1.3%)	3	3 (3.8%)
Dry skin	2	2 (2.5%)	1	1 (1.3%)	0	0 (0.0%)
Eczema	3	3 (3.8%)	2	2 (2.5%)	8	5 (6.3%)
Pain of skin	0	0 (0.0%)	1	1 (1.3%)	1	1 (1.3%)
Pruritus	1	1 (1.3%)	2	2 (2.5%)	0	0 (0.0%)
Rash acneiform	1	1 (1.3%)	0	0 (0.0%)	0	0 (0.0%)
Other, dermatitis	1	1 (1.3%)	0	0 (0.0%)	0	0 (0.0%)
Cut	1	1 (1.3%)	0	0 (0.0%)	0	0 (0.0%)
Hives	0	0 (0.0%)	0	0 (0.0%)	1	1 (1.3%)
Itching	1	1 (1.3%)	0	0 (0.0%)	0	0 (0.0%)
Rash	1	1 (1.3%)	1	1 (1.3%)	0	0 (0.0%)
Psoriasis	2	1 (2.6%)	0	0 (0.0%)	0	0 (0.0%)
Insect bite	1	1 (1.3%)	1	1 (1.3%)	1	1 (1.3%)
Sun burn	0	0 (0.0%)	0	0 (0.0%)	1	1 (1.3%)
Skin ulceration	1	1 (1.3%)	1	1 (1.3%)	0	0 (0.0%)
Urticaria	1	1 (1.3%)	0	0 (0.0%)	0	0 (0.0%)
**Surgical and medical procedures**	**11**	**10 (12.5%)**	**8**	**8 (10.0%)**	**6**	**6 (7.6%)**
**Vascular disorders**	**1**	**1 (1.3%)**	**11**	**7 (8.8%)**	**5**	**4 (5.1%)**
Conduction disorder	1	1 (1.3%)	1	1 (1.3%)	1	1 (1.3%)
Hot flashes	0	0 (0.0%)	2	2 (2.5%)	0	0 (0.0%)
Hypertension	0	0 (0.0%)	8	5 (6.3%)	4	4 (5.1%)

Data are presented as number (percentage).

© 2024 Gregori G et al. *JAMA Network Open.*

eTable 8. Analyses of the GSRS in the safety population.

Outcome variable	Visit	LS means (95% CI) p-value			Difference in LS means (95% CI) p-value	
		High-dose	Low-dose	Placebo	High-dose - Placebo	Low-dose - Placebo
**Change in GSRS Reflux score**	**Year 1**	-0.00 (-0.12; 0.11) p=0.96	0.04 (-0.07; 0.16) p=0.45	-0.03 (-0.15; 0.08) p=0.59	0.03 (-0.14; 0.19) p=0.73	0.08 (-0.09; 0.24) p=0.36
	**Year 2**	0.07 (-0.06; 0.20) p=0.28	0.06 (-0.07; 0.19) p=0.33	-0.05 (-0.18; 0.08) p=0.45	0.12 (-0.06; 0.30) p=0.19	0.11 (-0.07; 0.29) p=0.22
**Change in GSRS Abdominal Pain score**	**Year 1**	-0.10 (-0.22; 0.02) p=0.09	0.02 (-0.10; 0.14) p=0.78	0.06 (-0.06; 0.18) p=0.31	-0.16 (-0.33; 0.00) p=0.06	-0.04 (-0.21; 0.12) p=0.61
	**Year 2**	-0.01 (-0.13; 0.10) p=0.82	-0.07 (-0.18; 0.05) p=0.24	0.02 (-0.10; 0.13) p=0.74	-0.03 (-0.19; 0.13) p=0.69	-0.09 (-0.25; 0.08) p=0.29
**Change in GSRS Indigestion score**	**Year 1**	0.02 (-0.12; 0.16) p=0.82	-0.01 (-0.14; 0.13) p=0.94	-0.06 (-0.19; 0.08) p=0.41	0.07 (-0.12; 0.27) p=0.46	0.05 (-0.14; 0.25) p=0.60
	**Year 2**	-0.02 (-0.14; 0.11) p=0.78	0.07 (-0.06; 0.20) p=0.27	-0.07 (-0.19; 0.06) p=0.29	0.05 (-0.13; 0.23) p=0.58	0.14 (-0.04; 0.32) p=0.13
**Change in GSRS Diarrhea score**	**Year 1**	-0.05 (-0.18; 0.08) p=0.44	-0.03 (-0.16; 0.10) p=0.65	-0.02 (-0.15; 0.11) p=0.72	-0.03 (-0.21; 0.16) p=0.77	-0.01 (-0.19; 0.18) p=0.94
	**Year 2**	0.09 (-0.07; 0.25) p=0.29	0.04 (-0.13; 0.20) p=0.65	-0.05 (-0.22; 0.11) p=0.51	0.14 (-0.09; 0.37) p=0.23	0.09 (-0.14; 0.32) p=0.44
**Change in GSRS Constipation score**	**Year 1**	-0.08 (-0.23; 0.08) p=0.33	-0.05 (-0.20; 0.10) p=0.52	0.06 (-0.09; 0.22) p=0.41	-0.14 (-0.36; 0.08) p=0.20	-0.11 (-0.33; 0.10) p=0.30
	**Year 2**	-0.00 (-0.14; 0.14) p=0.99	-0.06 (-0.20; 0.08) p=0.42	0.10 (-0.03; 0.24) p=0.14	-0.10 (-0.30; 0.09) p=0.29	-0.16 (-0.36; 0.04) p=0.11
**Change in GSRS Total score**	**Year 1**	-0.04 (-0.12; 0.05) p=0.37	-0.01 (-0.09; 0.07) p=0.79	0.00 (-0.08; 0.08) p=0.98	-0.04 (-0.16; 0.08) p=0.52	-0.01 (-0.13; 0.10) p=0.84
	**Year 2**	0.03 (-0.05; 0.12) p=0.47	0.00 (-0.08; 0.09) p=0.96	-0.01 (-0.10; 0.08) p=0.82	0.04 (-0.08; 0.16) p=0.50	0.01 (-0.11; 0.13) p=0.84

Mixed models for repeated measures is applied with change from baseline as outcome variable, visit, treatment group, interaction visit*treatment group as main fixed effects and baseline value as covariate. Unstructured covariance pattern is used for correlated data repeated over time. Diagnostic plots of residuals were investigated and found satisfactory for indigestion, constipation and GSRS, and less good for reflux, abdominal pain, and diarrhoea.

© 2024 Gregori G et al. *JAMA Network Open.*

eTable 9. Exploratory analyses of the change in serum short chain fatty acids in the ITT population.

Variable	High-dose N=80	Low-dose N=80	Placebo N=79	p-value High-dose vs Placebo	p-value Low-dose vs Placebo
Change in Acetic acid from baseline to 1 year (%)	-22.5 (-95.3; 418.4) p=0.21	-12.9 (-81.9; 612.3) p=0.92	-6.79 (-87.9; 2190.7) p=0.65	0.18	0.53
Change in Acetic acid from baseline to 2 years (%)	-28.5 (-99.1; 196.9) p=<.0001	-24.9 (-91.4; 627.6) p=0.13	-5.23 (-91.4; 874.1) p=0.56	0.022	0.39
Change in Propionic acid from baseline to 1 year (%)	10.0 (-81.0; 204.0) p=0.049	9.12 (-81.25; 1020.0) p=0.049	12.2 (-88.32; 442.2) p=0.14	0.79	0.62
Change in Propionic acid from baseline to 2 years (%)	3.70 (-77.8; 361.3) p=0.81	-1.00 (-67.90; 732.3) p=0.63	-0.24 (-81.36; 188.4) p=0.79	0.63	0.86
Change in Butyric acid from baseline to 1 year (%)	2.47 (-96.6; 353.6) p=0.20	2.70 (-92.15; 459.8) p=0.79	-1.15 (-90.77; 450.4) p=0.73	0.94	0.98
Change in Butyric acid from baseline to 2 years (%)	3.00 (-76.8; 408.2) p=0.37	2.15 (-83.4; 471.6) p=0.73	-3.27 (-84.24; 551.8) p=0.37	0.34	0.68

Data are presented as mean±standard deviation, median (range), IQR and number of observations.
For test of the changes from baseline Wilcoxon Signed Rank test was used. For test between treatment groups with respect percent change from baseline Mann-Whitney U-test was used.
Analysis between high dose vs placebo and low dose vs placebo, defined in the SAP to be formally tested were PINP, CTX, butyric acid, acetic acid and propionic acid. Following Bonferroni-Holm adjustment none of the p-values for these variables were significant at 0.05 level.

© 2024 Gregori G et al. *JAMA Network Open.*

eTable 10. **Subgroup analyses of the primary efficacy variable (ITT population)**

			LS means (95% CI)			Difference in LS means (95% CI)		Interaction
**Outcome variable**		**Visit**	**High dose**	**Low dose**	**Placebo**	**High dose - Placebo**	**Low dose - Placebo**	**p-value**
**Percent change in tibia ultradistal (standard site) total volumetric BMD (%)**	**Years since menopause ≤ 2**	**Year 1**	-1.34 (-1.81; -0.86) n=53	-1.21 (-1.69; -0.73) n=50	-1.21 (-1.74; -0.69) n=42	-0.12 (-0.82; 0.58)	0.00 (-0.71; 0.71)	0.72
		**Year 2**	-2.85 (-3.56; -2.14) n=53	-2.91 (-3.64; -2.18) n=50	-2.48 (-3.27; -1.69) n=42	-0.37 (-1.43; 0.70)	-0.43 (-1.51; 0.65)	0.46
	**Years since menopause >2**	**Year 1**	-0.97 (-1.86; -0.08) n=27	-0.65 (-1.51; 0.22) n=30	-1.21 (-1.95; -0.46) n=37	0.23 (-0.93; 1.39)	0.56 (-0.59; 1.71)	
		**Year 2**	-1.52 (-2.37; -0.68) n=27	-1.88 (-2.70; -1.06) n=30	-2.14 (-2.86; -1.43) n=37	0.62 (-0.49; 1.72)	0.26 (-0.84; 1.36)	
	**BMI <25 kg/m^2**	**Year 1**	-1.84 (-2.46; -1.22) n=43	-1.31 (-1.91; -0.71) n=43	-1.40 (-1.99; -0.81) n=45	-0.44 (-1.29; 0.41)	0.09 (-0.75; 0.94)	0.28
		**Year 2**	-3.46 (-4.20; -2.72) n=43	-3.02 (-3.74; -2.30) n=43	-2.56 (-3.28; -1.85) n=45	-0.90 (-1.93; 0.13)	-0.46 (-1.47; 0.56)	0.043
	**BMI ≥ 25 kg/m^2**	**Year 1**	-0.47 (-1.08; 0.13) n=37	-0.67 (-1.30; -0.05) n=37	-0.94 (-1.55; -0.32) n=34	0.46 (-0.40; 1.32)	0.26 (-0.61; 1.13)	
		**Year 2**	-1.15 (-1.89; -0.40) n=37	-1.91 (-2.68; -1.14) n=37	-1.99 (-2.76; -1.23) n=34	0.85 (-0.22; 1.91)	0.09 (-1.00; 1.17)	
	**IPAQ <median METs/week**	**Year 1**	-0.84 (-1.34; -0.34) n=42	-0.97 (-1.49; -0.45) n=42	-1.07 (-1.61; -0.52) n=35	0.22 (-0.52; 0.96)	0.10 (-0.66; 0.85)	0.72
		**Year 2**	-2.20 (-2.93; -1.48) n=42	-2.24 (-2.99; -1.48) n=42	-2.02 (-2.81; -1.22) n=35	-0.19 (-1.26; 0.89)	-0.22 (-1.32; 0.87)	0.85
	**IPAQ ≥ median METs/week**	**Year 1**	-1.57 (-2.31; -0.82) n=38	-1.18 (-1.89; -0.47) n=38	-1.27 (-1.93; -0.61) n=44	-0.30 (-1.29; 0.70)	0.09 (-0.88; 1.06)	
		**Year 2**	-2.53 (-3.36; -1.69) n=38	-2.91 (-3.72; -2.11) n=38	-2.54 (-3.29; -1.78) n=44	0.01 (-1.12; 1.13)	-0.38 (-1.48; 0.73)	
	**CTX < median**	**Year 1**	-0.67 (-1.03; -0.31) n=45	-0.56 (-0.96; -0.15) n=37	-0.58 (-0.95; -0.21) n=40	-0.09 (-0.61; 0.42)	0.02 (-0.52; 0.57)	0.77
		**Year 2**	-1.64 (-2.16; -1.12) n=45	-1.78 (-2.37; -1.20) n=37	-1.61 (-2.14; -1.07) n=40	-0.03 (-0.78; 0.71)	-0.17 (-0.97; 0.62)	0.84
	**CTX ≥ median**	**Year 1**	-1.82 (-2.65; -1.00) n=35	-1.44 (-2.17; -0.72) n=43	-1.86 (-2.63; -1.09) n=39	0.04 (-1.09; 1.17)	0.42 (-0.64; 1.48)	
		**Year 2**	-3.27 (-4.23; -2.30) n=35	-3.16 (-4.02; -2.30) n=43	-3.07 (-3.98; -2.15) n=39	-0.20 (-1.53; 1.13)	-0.09 (-1.34; 1.16)	
	**Protein intake < median**	**Year 1**	-1.45 (-2.04; -0.87) n=48	-1.12 (-1.81; -0.42) n=33	-0.86 (-1.49; -0.23) n=38	-0.59 (-1.45; 0.27)	-0.26 (-1.20; 0.68)	0.14

© 2024 Gregori G et al. *JAMA Network Open.*

			LS means (95% CI)			Difference in LS means (95% CI)		Interaction
**Outcome variable**		**Visit**	**High dose**	**Low dose**	**Placebo**	**High dose - Placebo**	**Low dose - Placebo**	**p-value**
		**Year 2**	-2.87 (-3.62; -2.12) n=48	-2.46 (-3.35; -1.57) n=33	-2.16 (-2.97; -1.35) n=38	-0.71 (-1.82; 0.39)	-0.30 (-1.50; 0.91)	0.12
	**Protein intake ≥ median**	**Year 1**	-0.88 (-1.56; -0.21) n=32	-0.95 (-1.53; -0.37) n=47	-1.52 (-2.12; -0.91) n=41	0.63 (-0.27; 1.54)	0.56 (-0.27; 1.40)	
		**Year 2**	-1.74 (-2.55; -0.93) n=32	-2.56 (-3.26; -1.87) n=47	-2.46 (-3.19; -1.72) n=41	0.72 (-0.37; 1.81)	-0.11 (-1.12; 0.90)	
	**Calcium intake < median**	**Year 1**	-1.16 (-1.71; -0.61) n=47	-0.92 (-1.53; -0.31) n=39	-0.61 (-1.24; 0.03) n=33	-0.56 (-1.40; 0.29)	-0.31 (-1.19; 0.57)	0.20
		**Year 2**	-2.41 (-3.15; -1.68) n=47	-2.39 (-3.21; -1.58) n=39	-1.84 (-2.69; -0.99) n=33	-0.57 (-1.70; 0.55)	-0.55 (-1.73; 0.62)	0.35
	**Calcium intake ≥ median**	**Year 1**	-1.30 (-2.01; -0.60) n=33	-1.04 (-1.67; -0.41) n=41	-1.68 (-2.27; -1.09) n=46	0.38 (-0.54; 1.29)	0.64 (-0.22; 1.51)	
		**Year 2**	-2.38 (-3.21; -1.55) n=33	-2.56 (-3.31; -1.82) n=41	-2.70 (-3.40; -2.00) n=46	0.33 (-0.76; 1.41)	0.14 (-0.89; 1.16)	
	**Total hip BMD T-score <-1**	**Year 1**	-1.74 (-2.63; -0.85) n=21	-1.18 (-2.21; -0.15) n=15	-1.32 (-2.26; -0.38) n=19	-0.42 (-1.72; 0.88)	0.14 (-1.25; 1.54)	0.71
		**Year 2**	-2.98 (-4.19; -1.76) n=21	-2.83 (-4.23; -1.43) n=15	-2.84 (-4.12; -1.55) n=19	-0.14 (-1.91; 1.63)	0.01 (-1.90; 1.91)	0.95
	**Total hip BMD T-score** ≥-1	**Year 1**	-1.00 (-1.52; -0.49) n=59	-0.99 (-1.48; -0.50) n=65	-1.16 (-1.65; -0.66) n=60	0.15 (-0.56; 0.86)	0.16 (-0.53; 0.86)	
		**Year 2**	-2.16 (-2.78; -1.55) n=59	-2.45 (-3.04; -1.86) n=65	-2.15 (-2.74; -1.56) n=60	-0.01 (-0.87; 0.84)	-0.30 (-1.14; 0.53)	

Mixed models for repeated measures are applied with percent difference as outcome variable, visit, treatment group, interaction visit*treatment group as main fixed effects and baseline value as covariate. Unstructured covariance pattern is used for correlated data repeated over time. Diagnostic plots of residuals were investigated and found satisfactory.

© 2024 Gregori G et al. *JAMA Network Open.*

eTable 11. Logistic regression for responders based on the responders defined as percent change in total tibia vBMD at two years >-1.0 (all high- and low-dose participants included).

Variable	Values	Missing data	n (%) responders per value	OR (95% CI)	p-value	AUC
Age (years) (per 1 SD increase)	<=median	0	20 (19.0%)			
	>median	0	17 (30.9%)	1.35 (0.93 - 1.95)	0.11	0.59
Height (cm) (per 1 SD increase)	<=median	0	18 (22.5%)			
	>median	0	19 (23.8%)	1.09 (0.75 - 1.58)	0.66	0.52
Weight (kg) (per 1 SD increase)	<=median	0	12 (15.0%)			
	>median	0	25 (31.3%)	1.45 (1.02 - 2.06)	0.040	0.64
Body mass index (kg/m^2) (per 1 SD increase)	<=median	0	12 (15.0%)			
	>median	0	25 (31.3%)	1.50 (1.05 - 2.14)	0.028	0.65
Total body fat (%)(per 1 SD increase)	<=median	0	13 (16.3%)			
	>median	0	24 (30.0%)	1.72 (1.15 - 2.56)	0.008	0.65
Appendicular lean mass (kg/m^2) (per 1 SD increase)	<=median	0	16 (20.0%)			
	>median	0	21 (26.3%)	1.20 (0.84 - 1.73)	0.31	0.56
Current smoking (ref No)	No	0	37 (23.7%)			
	Yes	0	0 (0.0%)	0.00 (0.00 - I)	0.98	0.52
Parental hip fracture (ref No)	No	0	29 (20.7%)			
	Yes	0	8 (40.0%)	2.55 (0.95 - 6.82)	0.06	0.56
Run-in period (days) (per 1 SD increase)	<=median	0	22 (23.2%)			
	>median	0	15 (23.1%)	1.03 (0.71 - 1.49)	0.88	0.51
Previous glucocorticoids (5mg of Prednisolone or more for 3 months or more) (ref No)	No	0	35 (22.3%)			
	Yes	0	2 (66.7%)	6.96 (0.61 - 79.05)	0.12	0.52
Tibia ultradistal (standard site) Cortical area (mm) (per 1 SD increase)	<=median	0	18 (22.5%)			
	>median	0	19 (23.8%)	0.91 (0.63 - 1.32)	0.63	0.51
Tibia ultradistal (standard site) total volumetric BMD (mg/cm^3) (per 1 SD increase)	<=median	0	16 (20.0%)			
	>median	0	21 (26.3%)	1.20 (0.83 - 1.74)	0.33	0.55
Tibia ultradistal (standard site) cortical volumetric BMD (mg/cm^3) (per 1 SD increase)	<=median	0	16 (20.0%)			
	>median	0	21 (26.3%)	1.00 (0.69 - 1.45)	1.00	0.52
Tibia ultradistal (standard site) trabecular bone volume fraction (%) (per 1 SD increase)	<=median	0	14 (17.3%)			
	>median	0	23 (29.1%)	1.26 (0.88 - 1.80)	0.20	0.56
Bone mineral density (DXA) total hip (g/cm^2) (per 1 SD increase)	<=median	0	16 (20.0%)			
	>median	0	21 (26.3%)	1.25 (0.87 - 1.80)	0.23	0.56
Bone mineral density (DXA) femoral neck (g/cm^2) (per 1 SD increase)	<=median	0	17 (21.3%)			
	>median	0	20 (25.0%)	1.09 (0.76 - 1.57)	0.64	0.52
Bone mineral density (DXA) lumbar spine (g/cm^2) (per 1 SD increase)	<=median	0	21 (26.3%)			
	>median	0	16 (20.0%)	1.12 (0.78 - 1.61)	0.55	0.52
T-score total hip (SD) (per 1 SD increase)	<=median	0	16 (20.0%)			
	>median	0	21 (26.3%)	1.25 (0.87 - 1.80)	0.23	0.56
T-score lumbar spine (SD) (per 1 SD increase)	<=median	0	21 (26.3%)			
	>median	0	16 (20.0%)	1.12 (0.78 - 1.61)	0.55	0.52

© 2024 Gregori G et al. *JAMA Network Open.*

Variable	Values	Missing data	n (%) responders per value	OR (95% CI)	p-value	AUC
10-year fracture probability (FRAX) for major osteoporotic fracture (with BMD) (per 1 SD increase)	<=median	0	14 (17.5%)			
	>median	0	23 (28.8%)	1.56 (1.09 - 2.21)	0.014	0.61
10-year fracture probability (FRAX) for major osteoporotic fracture (without BMD) (per 1 SD increase)	<=median	0	16 (19.5%)			
	>median	0	21 (26.9%)	1.42 (1.00 - 2.00)	0.048	0.57
10-year fracture probability (FRAX) for hip fracture (with BMD) (per 1 SD increase)	<=median	0	17 (21.3%)			
	>median	0	20 (25.0%)	1.04 (0.72 - 1.49)	0.83	0.51
10-year fracture probability (FRAX) for hip fracture (without BMD) (per 1 SD increase)	<=median	0	19 (23.8%)			
	>median	0	18 (22.5%)	1.03 (0.72 - 1.49)	0.85	0.50
Serum 25-OH-vitamin D (nmol/l) (per 1 SD increase)	<=median	0	19 (23.2%)			
	>median	0	18 (23.1%)	0.88 (0.60 - 1.29)	0.50	0.52
Plasma total calcium (mmol/l) (per 1 SD increase)	<=median	0	23 (25.8%)			
	>median	0	14 (19.7%)	0.87 (0.60 - 1.26)	0.46	0.55
PINP (µg/L) (per 1 SD increase)	<=median	1	22 (27.5%)			
	>median	1	14 (17.7%)	0.90 (0.61 - 1.34)	0.62	0.58
CTX (ng/ml) (per 1 SD increase)	<=median	6	19 (24.7%)			
	>median	6	14 (18.2%)	0.77 (0.49 - 1.20)	0.25	0.55
GSRS Total score (per 1 SD increase)	<=median	1	18 (21.7%)			
	>median	1	19 (25.0%)	1.13 (0.80 - 1.59)	0.50	0.55
Energy intake (kcal/day) (per 1 SD increase)	<=median	0	20 (25.0%)			
	>median	0	17 (21.3%)	1.15 (0.81 - 1.63)	0.45	0.52
Protein intake (g/day) (per 1 SD increase)	<=median	0	18 (22.5%)			
	>median	0	19 (23.8%)	1.03 (0.72 - 1.48)	0.87	0.52
Fat intake (g/day) (per 1 SD increase)	<=median	0	21 (26.3%)			
	>median	0	16 (20.0%)	1.04 (0.73 - 1.50)	0.82	0.49
Carbohydrates intake (g/day) (per 1 SD increase)	<=median	0	17 (21.3%)			
	>median	0	20 (25.0%)	1.21 (0.86 - 1.71)	0.27	0.56
Fiber intake (g/day) (per 1 SD increase)	<=median	0	18 (22.5%)			
	>median	0	19 (23.8%)	1.14 (0.80 - 1.61)	0.47	0.51
Salt intake (g/day) (per 1 SD increase)	<=median	0	15 (18.8%)			
	>median	0	22 (27.5%)	1.11 (0.78 - 1.58)	0.55	0.54
Calcium intake (mg/day) (per 1 SD increase)	<=median	0	22 (27.5%)			
	>median	0	15 (18.8%)	0.90 (0.61 - 1.33)	0.60	0.53
Physical activity (IPAQ, METs per week) (per 1 SD increase)	<=median	0	20 (25.0%)			
	>median	0	17 (21.3%)	0.44 (0.18 - 1.13)	0.09	0.58
Years since menopause (per 1 SD increase)	<=median	0	20 (19.4%)			
	>median	0	17 (29.8%)	1.40 (0.97 - 2.02)	0.07	0.60
Calcium and vitamin D supplements taken 3 months prior to baseline (Ref No)	No	2	33 (22.3%)			
	Yes	2	3 (30.0%)	1.49 (0.37 - 6.10)	0.58	0.51

© 2024 Gregori G et al. *JAMA Network Open.*

eFigure. Pearson correlation between BMI and % change in tibia total vBMD by treatment group over time (ITT population).

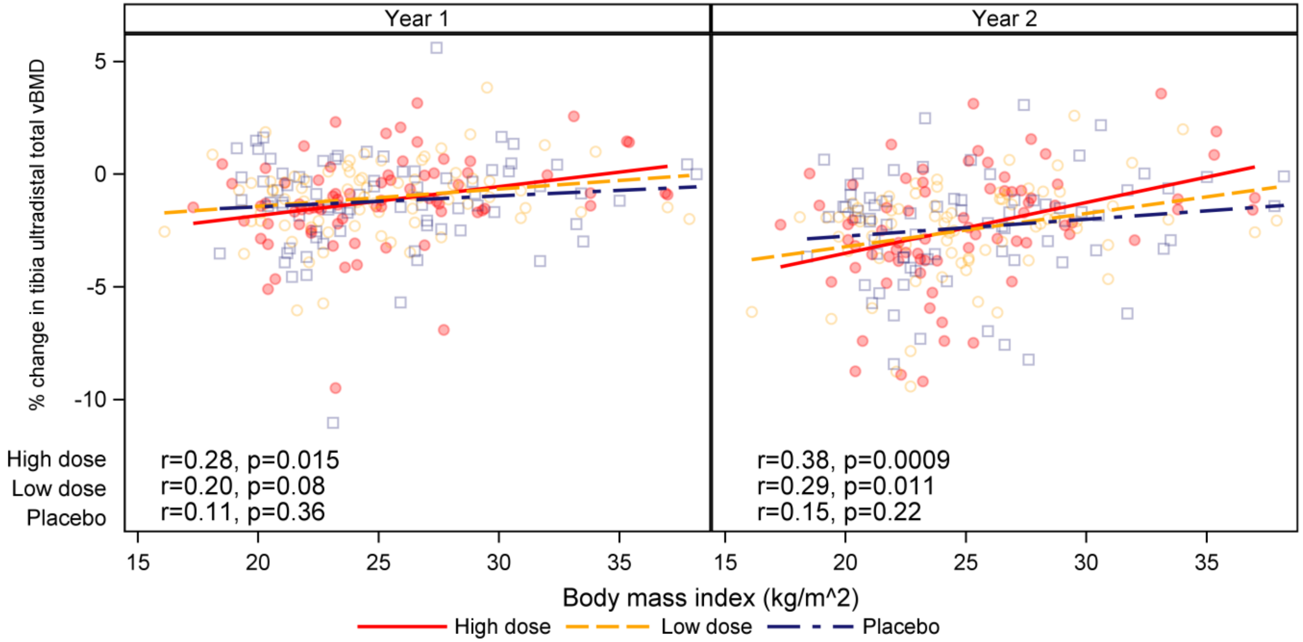

© 2024 Gregori G et al. *JAMA Network Open.*
