# Supplementary material for: Limosilactobacillus reuteri 6475 and Prevention of Early Postmenopausal Bone Loss: A Randomized Clinical Trial
Source: JAMA Netw Open. 2024 Jun 12;7(6):e2415455. doi: 10.1001/jamanetworkopen.2024.15455 (PMC11170297; doi:10.1001/jamanetworkopen.2024.15455)
Supplement: Supplement 4. — Data Sharing Statement [file jamanetwopen-e2415455-s004.pdf]

Data Sharing Statement

Gregori. Limosilactobacillus reuteri 6475 and Prevention of Early Postmenopausal Bone Loss. *JAMA Netw Open*. Published June 12, 2024. doi:10.1001/jamanetworkopen.2024.15455

Data
Data available: No

Additional Information
Explanation for why data not available: Data availability Data cannot be made publicly available for ethical and legal reasons. Such information is subject to legal restrictions according to national legislation. Specifically, in Sweden confidentiality regarding personal information in studies is regulated in the Public Access to Information and Secrecy Act (SFS 2009:400). The data underlying the results of this study might be made available upon request, after an assessment of confidentiality. There is thus a possibility to apply to get access to certain public documents that an authority holds. In this case, the University of Gothenburg is the specific authority that is responsible for the integrity of the documents with research data. Questions regarding such issues can be directed to the head of the Institute of Medicine, Sahlgrenska Academy, University of Gothenburg, Gothenburg, Sweden. Contact information can be obtained from medicin@gu.se.
